# Homoacetogenesis and microbial community composition are shaped by pH and total sulfide concentration

**DOI:** 10.1111/1751-7915.13546

**Published:** 2020-03-03

**Authors:** Eleftheria Ntagia, Ioanna Chatzigiannidou, Adam J. Williamson, Jan B. A. Arends, Korneel Rabaey

**Affiliations:** ^1^ Center for Microbial Ecology and Technology (CMET) Ghent University Coupure Links 653 Ghent 9000 Belgium

## Abstract

Biological CO_2_ sequestration through acetogenesis with H_2_ as electron donor is a promising technology to reduce greenhouse gas emissions. Today, a major issue is the presence of impurities such as hydrogen sulfide (H_2_S) in CO_2_ containing gases, as they are known to inhibit acetogenesis in CO_2_‐based fermentations. However, exact values of toxicity and inhibition are not well‐defined. To tackle this uncertainty, a series of toxicity experiments were conducted, with a mixed homoacetogenic culture, total dissolved sulfide concentrations ([TDS]) varied between 0 and 5 mM and pH between 5 and 7. The extent of inhibition was evaluated based on acetate production rates and microbial growth. Maximum acetate production rates of 0.12, 0.09 and 0.04 mM h^‐1^ were achieved in the controls without sulfide at pH 7, pH 6 and pH 5. The half‐maximal inhibitory concentration (IC_50_^qAc^) was 0.86, 1.16 and 1.36 mM [TDS] for pH 7, pH 6 and pH 5. At [TDS] above 3.33 mM, acetate production and microbial growth were completely inhibited at all pHs. 16S rRNA gene amplicon sequencing revealed major community composition transitions that could be attributed to both pH and [TDS]. Based on the observed toxicity levels, treatment approaches for incoming industrial CO_2_ streams can be determined.

## Graphical abstract

Biological CO_2_ sequestration through acetogenesis is a promising technology to reduce greenhouse gas emissions. However, a major issue is the presence of impurities such as hydrogen sulfide (H2S). A series of toxicity experiments were conducted, with a mixed homoacetogenic culture, total dissolved sulfide concentrations ([TDS]) varied between 0 and 5 mM and pH between 5 and 7. At [TDS] above 3.33 mM, acetate production and microbial growth were completely inhibited at all pHs. 16S rRNA gene amplicon sequencing revealed major community composition transitions that could be attributed to both pH and [TDS].

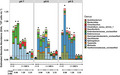

## Introduction

Carbon dioxide (CO_2_) emitted from industrial activities can be utilized by reduction into commodity chemicals within a general carbon capture and utilization (CCU) scheme. One such avenue of CO_2_ utilization is through gas fermentations, where homoacetogenic bacteria are employed to convert CO_2_ to acetate, using hydrogen (H_2_) as electron donor, through the Wood–Lungdahl pathway, which allows for linear CO_2_ fixation (Drake *et al.*, 2008). This pathway allows acetogenic bacteria to grow on C1 substrates, which signifies the importance of these bacterial cultures for industrial biotechnology applications. Starting with the isolation of *Clostridium aceticum* (Wieringa, 1939)*,* more than 100 acetogenic species have been isolated to date. A detailed overview of the acetogenic communities, the mechanism of the Wood–Lungdahl pathway and gas fermentation operational perspectives is given in several reviews (Drake *et al.*, 2008; Liew *et al.*, 2016).

Gas emissions from point sources by some of the largest CO_2_ emitting industries, such as petroleum refineries (Perez, 2013; Vostrikov *et al.*, 2017), steel (Mochizuki and Tsubouchi, 2017), pulp and paper (de Souza, 1988) and power production industry (particularly geothermal) (Kristmannsdóttir *et al.*, 2000), as well as biogas production, are often accompanied by various impurities (Osorio and Torres, 2009; Vostrikov *et al.*, 2017). A major concern when upgrading these gas streams in the context of CCU is that H_2_S, as one of the more common impurities, can already be toxic for microorganisms, both in pure culture and within microbial communities, at concentrations of a few ppm (Wu *et al.*, 2015). Several strategies for H_2_S removal exist (Mandal *et al.*, 2004; Pikaar *et al.*, 2015; Vaiopoulou *et al.*, 2016), but these processes will only lower the concentration of H_2_S and a fraction of it will inevitably end up in a gas fermentation reactor, where it can affect the microbial activity. The amount of H_2_S in the fermentation reactor will be dependent on the prior H_2_S removal steps (Kristmannsdóttir *et al.*, 2000). These steps will increase the total process costs, which can be avoided if we achieve a better understanding of the inhibitory effect and extent of certain impurities (Liew *et al.*, 2016). Complete removal is also not desirable as anaerobic microorganisms require sulfide as nutrient (Dhar *et al.*, 2012).

Sulfide toxicity has been reported for both mammalian and bacterial cells, and the mechanisms of toxicity may range from a general inhibition of respiratory activity (Chen *et al.*, 2008; Bouillaud and Blachier, 2011), DNA damage and protein denaturation (Wu *et al.*, 2015) to inhibition of specific activities, unique for specific organisms. Sulfide impairs a number of specific metabolic activities such as anammox and denitrification, as well as sulfate reduction, by decreasing the haem c content (Jin *et al.*, 2013), by inhibiting the N_2_O reductase activity (Pan *et al.*, 2013) and the sulfur reductase activity of cytochrome c3 (Reis *et al.*, 1992) respectively. In the case of homoacetogenic bacteria, the information provided include either studies on CO‐utilizing acetogens, usually employed in synthesis gas conversion (Vega *et al.*, 1990; Grethlein *et al.*, 1992), or homoacetogens as part of a general anaerobic community active during anaerobic digestion (Colleran *et al.*, 1998; O’Flaherty *et al.*, 1998b; Dar *et al.*, 2008). Importantly, the primary focus of studies to date has been on neutral to alkaline pH systems, thus have not yet considered lower pH systems (pH 5‐pH 6) typical of CO_2_‐fed fermentations that aim for steering the bioproduction to higher value products, such as ethanol (Liew *et al.*, 2016). Furthermore, these studies rarely consider actual sulfide concentrations in solution during their activity tests; thus, the inhibitory sulfide concentrations are often misestimated (McCartney and Oleszkiewicz, 1991; Chen *et al.*, 2008). Homoacetogens are a highly versatile group of bacteria that thrive in both acidic and alkaline pH environments (Drake *et al.*, 2008) with a growth optimum between pH 5.5 (Grimalt‐Alemany *et al.*, 2018) and neutral (Braun and Gottschalk, 1982; Ayudthaya *et al.*, 2018; Grimalt‐Alemany *et al.*, 2018).

The extent of sulfide inhibition is expected to be determined by the operational pH applied in a fermentation reactor, since this directly affects the sulfide speciation (Lewis, 2010) as well as the microbial activity. It remains unclear whether hydrogen sulfide (H_2_S) or the bisulfide ion (HS^‐^) is responsible for the toxicity effect (Küster *et al.*, 2005; Chen *et al.*, 2008). However, there is a general consensus that the undissociated H_2_S molecule can more easily penetrate the bacterial cell membrane, diffuse in the cell (Küster *et al.*, 2005; Saad *et al.*, 2017) and hinder the bacterial metabolic processes. Nevertheless, O’Flaherty et al. reported that the concentration of the undissociated H_2_S molecule [H_2_S] was related to inhibition at the lower tested pH (6.8–7.2) and total dissolved sulfide concentration [TDS] at a pH above 7.2 (O’Flaherty *et al.*, 1998b). Information on the operational pH is rarely reported, making it impossible to reach a valid conclusion on the effective inhibitory concentrations (Chen *et al.*, 2008). It is also not known whether there are large differences between bacterial acetogenic species in terms of sensitivity.

Given the above, it is of importance to assess the tolerance of gas fermenting microbial communities and pure cultures for their response to sulfide concentration and pH‐driven speciation. In this work, a series of toxicity experiments were conducted in serum flasks, inoculated with a mixed homoacetogenic microbial community and exposed to a range of sulfide concentrations, from 0 up to 5 mM [TDS]. The pH values selected for hydrogenotrophic homoacetogenic growth ranged between 5 and 7. Inhibition was evaluated based on acetate production by the microbial community and biomass growth. The pH, sulfide concentration in liquid and gas phase and the partial pressure in each serum flask were closely monitored. The half‐maximal inhibitory concentration (IC_50_) of the mixed homoacetogenic community was subsequently calculated at three different pH levels (7, 6 and 5), thus providing a data set of inhibitory TDS/ H_2_S_(aq)_/HS^‐^ concentrations that has been lacking till now. This data set provides tolerable levels of TDS (= sum of H_2_S_(aq)_ and HS^‐^) and the individual influence of bisulfide (HS^‐^) and dissolved hydrogen sulfide (H_2_S_(aq)_) for future development of microbial CO_2_ conversion schemes, starting from waste gasses.

## Results

### Sulfide concentration induces inhibition of microbial growth and homoacetogenic acetate production

**Fig. 1:**
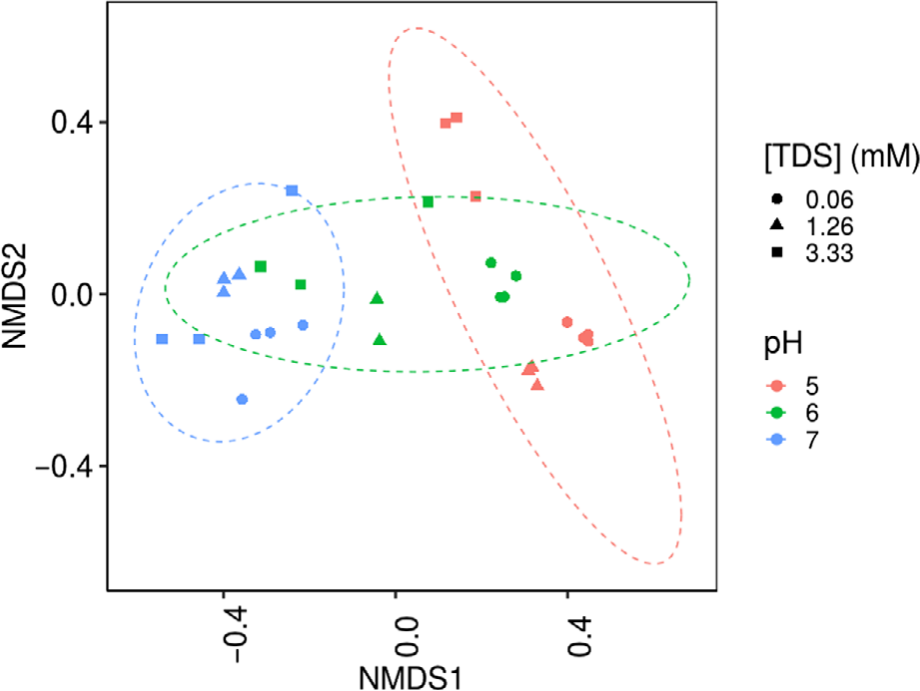
Non‐metric multidimensional scaling (NMDS) presenting the β‐diversity between different samples, calculated for *n* = 3 biological replicates, showing community dissimilarities with the pH shifts. A 95% confidence ellipse is drawn.

To ensure sulfide was the primary source of inhibition on the communities, the microbial community was acclimated prior to the experiments at pH 7, pH 6 and pH 5 (Fig. S1). The inoculation of every serum flask was done with an equal initial cell concentration, average of 6.54 ± 0.58 × 10^7^ cells ml^−1^ (Fig. S2). The preconditioning induced differences within the initial microbial community (Fig. 1). The β‐diversity NMDS plot revealed two distinct clusters for the samples grown in pH 5 and pH 7, whilst the pH 6 samples appeared between the two clusters. The inhibition results are discussed based solely on [TDS] (defined as the sum of the HS^‐^ and H_2_S_aq_), as these sulfide species will be present in the liquid phase, and as a function of pH (sulfide speciation).

**Fig. 2:**
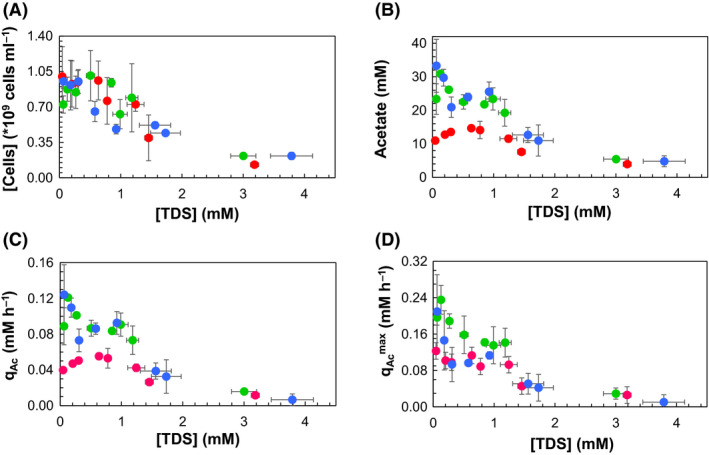
Plotted against initial total dissolved sulfide concentration ([TDS]) (mM) are (A) total cell concentration ([Cells], ×10^9^ cells ml^−1^), (B): final acetate concentration (mM), (C): overall acetate production rate (q_Ac_, mM h^‐1^) and (D): maximum acetate production rate (q_Ac_^max^, mM h^‐1^), calculated on a 48 h basis, at pH 7 (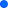), 6 (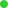) and 5 (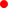) respectively. Data are averages of three incubations, and error bars represent standard deviations of biological triplicates. Complementary data are given in Figs S2, S5 and S6.

Sulfide inhibition was initially assessed through microbial growth. Prior to inoculation (t0), sulfide augmentation resulted in turbidity in the media, particularly at higher sulfide concentrations. This could represent some sulfide oxidation with residual oxygen and/or colloidal sulfide–metal complexes with the trace elements present in the medium (Table S4). Sulfide concentrations remained stable after addition, confirming no oxidation had occurred over time. Direct cell counts were thus assessed using flow cytometry (FCM), rather than optical density measurements. The biomass production in all pHs tested was significantly (*P* value < 0.05) impaired by increasing [TDS] (Fig. 2A and S4). For pH 7, the difference in total cell counts between low and high sulfide additions was 7.37 ± 0.45 × 10^8^ cells ml^−1^ (Fig. 2, Table S5) and the IC_50_^growth^ was 0.90 mM [TDS], 0.55 mM [H_2_S_aq_] and 0.34 mM [HS^‐^] (Table 1). At pH 6, the total cell counts differed by 5.08 ± 0.94 × 10^8^ cells ml^−1^ (Fig. 2A, Table S5) and the IC_50_^growth^ was 1.33, 1.15 and 0.17 mM [TDS], [H_2_S_aq_] and [HS^‐^] respectively (Table 1). At pH 5, the greatest difference (8.71 ± 2.91 × 10^8^ cells ml^−1^) in total cell counts between low and high sulfide additions was observed (Fig 2., Table S5) and the IC_50_^growth^ was 1.29 mM [TDS], 1.05 mM [H_2_S_aq_] and 0.07 mM [HS^‐^] (Table 1).

**Table 1:** Summary of the inhibition values (total inhibition and IC_50_ for overall and maximum acetate production rates (IC_50_^qAc^ and IC_50_^qAc_max^) and biomass growth based on FCM analysis (IC_50_^growth^). All IC_50_ values are reported in mM and are given as mean values and below, in brackets, the range of IC_50_ values, as calculated by GraphPad Prism 6 with a 95% confidence interval (CI).

	Inhibitor (mM)	Total inhibition	IC_50_^qAc^	IC_50_^qAc_max^	IC_50_^growth^
pH 7	TDS	3.79 (3.45–4.13)	0.86 (0.58–1.24)	0.44 (0.24–0.76)	0.90 (0.67–1.20)
	H_2_S	1.96 (1.87–2.05)	0.51 (0.34–0.73)*	0.27 (0.15–0.46)*,**	0.55 (0.43–0.69)*
	HS^‐^	1.82 (0.96–2.68)	0.34 (0.22–0.49)*	0.17 (0.09–0.29)	0.34 (0.24–0.51)*
pH 6	TDS	3.00 (2.80–3.21)	1.16 (0.84–1.63)	0.92 (0.66–1.27)	1.33 (1.01–2.16)
	H_2_S	2.37 (2.16–2.58)	1.01 (0.74–1.40)	0.81 (0.59–1.10)*	1.15 (0.90–1.76)
	HS^‐^	0.64 (0.62–0.66)	0.14 (0.09–0.23)	0.10 (0.07–0.16)	0.17 (0.11–0.39)
pH 5	TDS	3.19 (3.12–3.25)	1.36 (1.23–1.57)	1.16 (0.80–1.61)	1.29 (1.00–1.71)
	H_2_S	2.81 (2.75–2.87)	1.11 (1.00–1.27)*	0.98 (0.77–1.21)**	1.05 (0.88–1.29)*
	HS^‐^	0.37 (0.34–0.10)	0.08 (0.06–0.10)*	0.06 (0.03–0.10)	0.07 (0.05–0.12)*

All values are results of biological triplicates. Significant difference between inhibitors at different pH levels is noted on the table with ‘*’ and ‘**’, calculated based on two‐sample *t*‐test with equal variances (samples with *P* value <0.05 were considered significantly different).

The overall acetate production rate (q_Ac_) was selected as the primary indicator of microbial activity (Fig. 2). Complete inhibition of microbial activity (<0.02 mM h^‐1^ q_Ac_ and lowest final acetate concentration achieved) was observed at all pH values at the highest tested [TDS] (averaged [TDS] over all pH conditions = 3.33 ± 0.34 mM) (Table 1, Fig. 2B, C and S4). At all pH conditions, the optimum overall acetate production rates were observed in the lowest sulfide amended systems. At pH 7, the highest overall acetate production rate (Eq. 1) (q_Ac_) (0.12 ± 0.03 mM h^‐1^) was achieved. The IC_50_^qAc^ at pH 7 was calculated as 0.86 mM [TDS], 0.51 mM [H_2_S_aq_] and 0.34 mM [HS^‐^] (Table 1). At pH 6, a lower (0.09 ± 0.02 mM h^‐1^) q_Ac_ was achieved at the lowest sulfide addition (Fig. 2C, Table S5) and the IC_50_^qAc^ values were 1.16 mM [TDS], 1.01 mM [H_2_S_aq_] and 0.14 mM [HS^‐^] (Table 1). At pH 5, the lowest rates were reached, with 0.04 ± 0.01 for highest q_Ac_, with a corresponding IC_50_^qAc^ of 1.36 mM [TDS], 1.11 mM [H_2_S_aq_] and 0.08 mM [HS^‐^] (Table 1).

The difference between overall and maximum acetate production rates is critical for scale‐up operations of CO_2_ capture by homoacetogenic communities, since it will affect the fermentation reactor sizing and operation. In contrast to the overall acetate production rate as discussed above, the maximum acetate production rate (q_Ac_^max^) was calculated every 48 h of the experimental period. Similarly to q^Ac^, the q_Ac_^max^ decreased with decreasing pH at the lowest sulfide addition and at the highest sulfide addition the metabolic response was limited (<0.02 mM h^‐1^ q_Ac_^max^ at [TDS] = 3.33). The IC_50_ of q_Ac_^max^ increased with decreasing pH trend but with lower absolute values compared with IC_50_^qAc^ (Table 1). At pH 7, the q_Ac_^max^ difference achieved by the bacteria between the lowest and the highest sulfide addition was ~ 0.21 mM h^‐1^ (Fig. 2D, Table S5). The IC_50_^qAc_max^ was 0.44 mM [TDS], 0.27 mM [H_2_S_aq_] and 0.17 mM [HS^‐^], almost half of the IC_50_^qAc^ (Table 1). At pH 6, the difference between the highest and lowest q_Ac_^max^ was ~ 0.18 mM h^‐1^ (Fig. 2D, Table S5) and the IC_50_^qAc_max^ was calculated as 0.92, 0.81 and 0.10 mM [TDS], [H_2_S_aq_] and [HS^‐^] respectively (Table 1). Finally, at pH 5 the aforementioned difference was calculated as ~ 0.09 mM h^‐1^ and the IC_50_^qAc_max^ was 1.16, 0.98 and 0.06 mM [TDS], [H_2_S_aq_] and [HS^‐^] respectively (Table 1).

### Microbial community shifts in response to pH and sulfide concentration

**Fig. 3:**
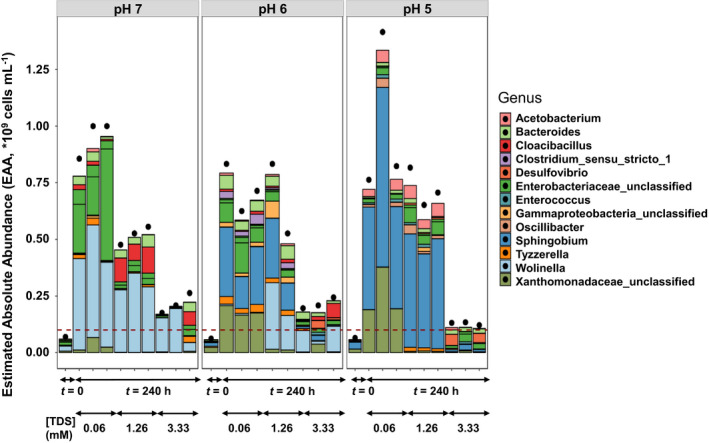
Estimated absolute abundances (EAAs) (cells ml^−1^) of the 15 most abundant OTUs, calculated as relative abundances normalized for the flow cytometric counts, in three biological replicates [and in two biological replicates at pH 6 and 1.26 mM (TDS)]. Black dots indicate the sum of EAA of all the OTUs identified with 16S rRNA gene amplicon sequencing analysis. The red dashed line represents the limit of 10^8^ cells ml^−1^. Non‐normalized relative abundances are shown in Fig. S7.

To understand whether an increase in biomass production was due to selective genera or a full community response, FCM was coupled to Illumina sequencing to estimate the absolute abundance (EAA) of individual community members (Fig. 3, S2, S7). Overall, the EAA of the top 15 genera present in the microbial community and presented here decreased with increased sulfide concentrations (Fig. 3 and S7). At pH 7, *Wolinella* was the most abundant genus recovered in the sequencing data. The EAA of this genus decreased significantly (*P* value = 0.008 < 0.05) by 3.0 ± 0.3 × 10^8^ cells ml^−1^, between 0.06 and 3.33 mM [TDS] (Fig. 3). At pH 6, the most abundant genus was *Sphingobium*, with a significant (*P* value = 0.011 < 0.05) EAA decrease of 2.2 ± 0.8 × 10^8^ cells ml^−1^ between highest and lowest [TDS] (Fig. 3). At pH 5, *Sphingobium* was also the most abundant genus and again the EAA decreased significantly (*P* value = 0.008 < 0.05) by 5.5 ± 1.5 × 10^8^ cells ml^−1^ when the [TDS] was increased to 3.33 mM.

The most abundant genus that is known for homoacetogenesis recovered in this study was *Acetobacterium* (Balch *et al.*, 1977). The EAA of the *Acetobacterium* at time 0 was similar for pH 7 and pH 6, with 8.23 × 10^5^ and 6.61 × 10^5^ cells ml^−1^, respectively, but higher (3.94 × 10^6^ cells ml^−1^) for pH 5, although the overall acetate production in this case was the lowest among all pH levels. At the lowest addition of sulfide, the EAA of *Acetobacterium* increased with decreasing pH; however, this genus accounted for less than 10% of the total relative abundances under all pH conditions and sulfide additions (Fig. 3).

**Fig. 4:**
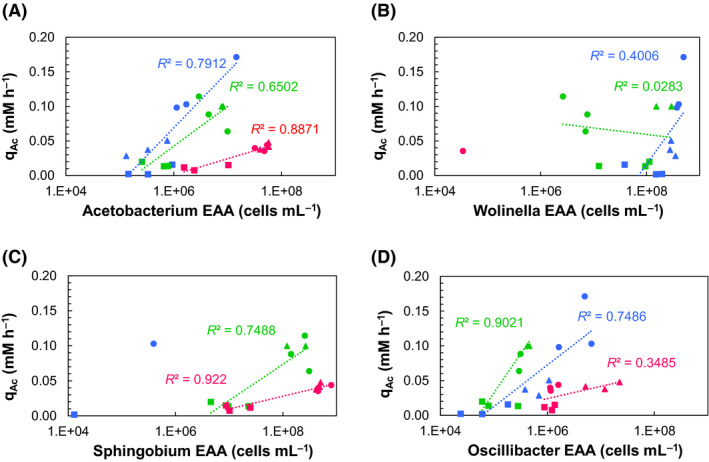
Overall acetate production rate (q_Ac_ in mM h^‐1^) correlated with the estimated absolute abundances (EAAs) (cells ml^−1^) of the genera (A): *Acetobacterium*, (B): *Wolinella*, (C): *Sphingobium* and (D): *Oscillibacter* at pH 7 (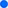), 6 (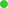) and 5 (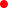), respectively, and at 0.06 (●), 1.26 (▲) and 3.33 (■) mM [TDS].

In the pH 7 incubation, the *Acetobacterium* EAA decreased gradually with increasing sulfide concentrations from an average of 5.8 ± 6.2 × 10^6^ cells ml^−1^ at 0.06 mM [TDS] to 4.7 ± 3.4 × 10^5^ cells ml^−1^ at 3.33 mM [TDS] (Fig. 3 and Fig. S3). At pH 6, the same trend was observed, with a decrease in EAA from an average of 5.8 ± 3.0 × 10^6^ cells ml^−1^ at 0.06 mM [TDS] to 5.7 ± 2.3 × 10^5^ cells ml^−1^ at 3.33 mM. At pH 5, the *Acetobacterium* EAA dropped significantly (*P* value = 0.005 < 0.5) from 4.5 ± 0.9 × 10^7^ to 4.8 ± 4.0 × 10^6^ cells ml^−1^ at 0.06 and 3.33 [TDS] respectively. In the pH 6 and pH 5 incubations at 1.26 mM [TDS], the abundance was higher than the one at 0.06 mM [TDS], suggesting that there is an optimal concentration of [TDS] for *Acetobacterium* species. This needs to be further explored in pure culture studies. The functional role of *Acetobacterium* in acetate production can be deduced from the correlation of EAA with final acetate concentrations (Fig. 4A) indicated by the production of acetate as the sole metabolic product. An analogous correlation can be found for the genera *Sphingobium* and *Oscillibacter* (Fig. 4C, D), but not for *Wolinella* (Fig 4B), although it should be mentioned here that any correlation in the case of genera that are not known for homoacetogenic bioproduction does not indicate a direct involvement of those genera in homoacetogenesis.

It is important to note that methane production was only observed at pH 7. This could be an indication of pH effect at first, considering that the majority of methanogens thrive in neutral up to slightly alkaline environments (Liu and Whitman, 2008). Nevertheless, at pH 7, a clear sulfide toxicity effect on methanogens was observed, with only minor methane production detected at 192 h (final [CH_4_] detected was 0.03 mM CH_4_ at 240 h) and only for the lowest [TDS] (0.06 ± 0.01 mM).

## Discussion

### Acetate production rates show higher sulfide sensitivity compared with biomass growth

In this study, the extent of sulfide toxicity on the growth and metabolic activity of a mixed homoacetogenic microbial community was examined at pH 5, pH 6 and pH 7. This allowed for quantification of the maximum [TDS], [H_2_S_aq_] and [HS^‐^] conducive to growth, as well as the IC_50_, inhibitory levels for homoacetogenic activity under both neutral and acidic conditions, data missing so far from literature. Overall, sulfide toxicity had a more profound effect on the rate of acetogenesis compared with total biomass growth, since the IC_50_^growth^ was higher than both the IC_50_^qAc^ and IC_50_^qAc_max^, in all pH levels tested (Table 1). In general, the TDS IC_50_ of all responses (growth, q_Ac_ and q_Ac_^max^) increased with decreasing pH, suggesting a higher tolerance in low pH homoacetogenic communities. The observation that the perceived weaker performing homoacetogenic community (lowest q_Ac_ at pH 5) displayed a higher resistance to sulfide is an interesting one. Typically, weaker systems are more susceptible to environmental stresses such as sulfide. The opposite was observed here; hence, the [TDS] as inhibitor could be less impactful under these already limited conditions.

The impact on the q_Ac_^max^ was more profound with increasing pH; at pH 7, the IC_50_^qAc_max^ was half of the IC_50_^qAc^ and IC_50_^growth^ (0.44 vs. 0.86 and 0.90, respectively, when defined with [TDS]), whereas at pH 5 they are similar (1.16 vs. 1.36 and 1.29, respectively, when defined with [TDS]). This was also supported by a longer lag phase in pH 5, compared to 7, observed in the incubations. The mechanisms of the different sulfide species that inhibited the cell growth and activity in this study are currently unknown, and may represent a combination of parameters governed by the equilibrium of HS^‐^/H_2_S_aq_ inside and outside of the bacterial cell (Howsley and Pearson, 1979) together with the obvious direct impact of pH. This difference in inhibition at different pH levels might indicate differentiation in environmental adaptation (Lloyd *et al.*, 2005). The lower IC_50_ (defined with [TDS]) at pH 7 could be associated with increased susceptibility to the H_2_S (Koster *et al.*, 1986), or conversely the higher IC_50_ at pH 5 could be associated with a decreased exposure to HS^‐^.

Growth inhibition and reduced activity of different anaerobic microbial populations under sulfide stress, including sulfate reducing, methanogenic and some syntrophic and fermentative bacteria, have been reported in operational results of anaerobic systems (Colleran *et al.*, 1998; O’Flaherty *et al.*, 1998a; Dar *et al.*, 2008). More specifically, the inhibition of methanogenesis by sulfide is already well described in literature (Koster *et al.*, 1986; McCartney and Oleszkiewicz, 1991; Chen *et al.*, 2008), and it has been reported that methanogens are more susceptible to sulfide toxicity than acetogens (Grimalt‐Alemany *et al.*, 2018). The results of the present study are in accordance with the results of McCartney and Oleszkiewicz (McCartney and Oleszkiewicz, 1991) where by testing anaerobic digestion of lactate and acetate under sulfide stress they observed that higher pH values and longer incubation times favoured methane production, whilst at lower pH values, methane production was decreasing with increasing total sulfide (TS) concentrations.

Despite the toxicity effect, sulfide is a molecule essential for the survival of the bacterial cells, preventing them from oxidative stress (Wu *et al.*, 2015) or metal toxicity (Lloyd *et al.*, 2005) whilst also an essential macronutrient for the survival of most anaerobic organisms (Dhar *et al.*, 2012). In any case, it appears that the mere H_2_S concentration cannot be used as sole discriminant towards inhibition.

### The microbial community shifts with sulfide concentration and speciation

Whilst pH was the primary driver of initial community composition (Fig. 1), the subsequent addition of sulfide had a clear impact on the community structure and EAA (Fig. 3). A decrease in the total cell numbers was observed in all pH incubations at high [TDS], accompanied with an increase in the community diversity (Figs 2A and 3). Prior to sulfide addition, *Wolinella* was the most abundant genus at pH 7, whereas at pH 6 and pH 5 the genus *Sphingobium* was dominant (Fig. 3). Nevertheless, *Wolinella* and *Sphingobium* are not classified in the class of Clostridia, to which most of the known homoacetogens belong (Drake *et al.*, 2008).

The presence of *Acetobacterium*, a known homoacetogen, is not surprising and has been observed in many studies involving pure homoacetogenic as well as mixed culture studies for CO_2_ reduction (Kantzow *et al.*, 2015; Liew *et al.*, 2016; Arends *et al.*, 2017). The relatively low relative abundances measured (~ 10%) in this study indicate that other species might be additionally responsible for homoacetogenic bioproduction. Interestingly, *Acetobacterium* was the only genus that showed a similar EAA at 0.06 and 1.26 mM [TDS] (Fig. 3) at pH 6 and pH 5, which suggests a higher tolerance to sulfide. Further linking of this genus to the IC_50_ values is not possible, as inhibition could be due to individual toxicity or synergistic community effects. Low concentrations of sulfide (~ 0.5 mM) have been reported to facilitate the growth of *Acetobacterium* on heterotrophic substrates (Heijthuijsen and Hansen, 1989); however, the influence of this higher sulfide concentration on this genus growing on H_2_/CO_2_ clearly warrants further investigation.

In order to be able to compare with literature data regarding the specific activity of *Acetobacterium*, an assumption was made for the cell dry weight of the community as previously described (Demler and Weuster‐Botz, 2011; Kantzow *et al.*, 2015). The highest specific activity calculated for *Acetobacterium* was approximately 33 g_acetate_/ g_CDW_ d, obtained at pH 7 and at 1.26 mM [TDS] in this study. This value is higher than the highest reported metabolic activity (20 g_acetate_/ g_CDW_ d) of the *Acetobacterium* genus (Straub *et al.*, 2014; Kantzow *et al.*, 2015) which reinforces that it is likely that *Acetobacterium* was not the only active acetogen in the mixed culture studied here. The hypothesis of heterotrophic acetate production can be abandoned based on a number of observations proving homoacetogenic activity. More specifically, (i) H_2_ and CO_2_ were consumed from the headspace, indicating autotrophic CO_2_ fixation, (ii) acetate was the sole product of acetogenesis, and (iii) the biomass concentration increased during the incubation, which serves as a counterargument for necrotrophic growth.

*Wolinella* was highly abundant in the homoacetogenic cultures at pH 6 and pH 7; however, little is known about its metabolic capacities, and in the incubations studied here, no correlation of the *Wolinella* EAA with acetate production was possible (Fig. 4B). Based on existing literature, its presence could be explained by their ability (i) to gain energy through polysulfide respiration with H_2_ as electron donor (Hedderich *et al.*, 1999; Dietrich and Klimmek, 2002) or (ii) through elemental sulfur respiration (Ringel *et al.*, 1996). Whilst no chemical analysis of elemental sulfur or polysulfides was conducted in our study, the sulfide concentration remained stable over the 240 h incubations. Furthermore, Fe^2+^ has been demonstrated to be crucial in this process; however, Fe^2+^ was four orders of magnitude lower in our system than in Ringel *et al.* (1996); thus, such processes appear unlikely (Table S4). The role of *Wolinella* as a H_2_ scavenger in co‐cultures with fermentative anaerobic bacteria has been described before (Cord‐Ruwisch *et al.*, 1988; Parameswaran *et al.*, 2010); thus, in the current study, syntrophic growth of *Wolinella* on H_2_ could be also the most probable association. Total growth inhibition of *Wolinella* by sulfide has been previously reported in co‐culture with *Geobacter* at ~ 1 mM added sulfide (Kaden *et al.*, 2002), whilst in this study, a higher tolerance to sulfide was observed (~ 28% decrease in cells at 1.33 mM), with growth inhibition occurring only at the highest sulfide concentration (~ 3 mM) [TDS] (Fig. 3).

The genus of *Sphingobium* has been reported to contain mostly aerobic and facultative anaerobic soil bacteria (Ushiba *et al.*, 2003; Singh and Lal, 2009; Berney *et al.*, 2014; Chaudhary *et al.*, 2017; Esposti and Romero, 2017). It has also been reported to perform sulfur respiration (Xia *et al.*, 2017), i.e. capable of producing H_2_S through reduction in organosulfur compounds and oxidizing this self‐produced H_2_S, under aerobic conditions. Since the current study was performed in anaerobic conditions, a clone library was made from the sample with the most abundant community of *Sphingobium* to confirm the sequencing results (S1.2 and S2.1). From the clone library, 25% of sequences were identified as *Sphingobium* with 96‐97% identity (S2.1). This limited sequence similarity most likely indicates a novel genus in this case. Although there are no metabolic data available yet that can explain the presence of this genus in an anaerobic system performing homoacetogenic production, in our incubation a correlation of its EAA with the acetate concentration, especially at pH 5 (Fig. 4C), was observed. It should be mentioned again that any correlation in this case indicates a plausible mechanism but not causation. Another possible explanation for its presence could be that it functions in organosulfur compound metabolism (Aylward *et al.*, 2013). The metabolic capabilities of the *Wolinella* and *Sphingobium* species recovered in this study and under these conditions should be further explored using pure culture or isolates.

### Technological implications and future perspectives

In this study, we determined the impact of sulfide on anaerobic homoacetogenic microbial consortia incubated at pH 5, pH 6 and pH 7. A typical biogas stream produced by anaerobic digestion of wastewater treatment plant sludge may contain up to 2000 ppmv H_2_S (Osorio and Torres, 2009), whereas other feedstocks for anaerobic digestion can lead to 30 000 ppmv H_2_S in the biogas (Barrera *et al.*, 2013). In our study, maximum acetate production rates (q_Ac_^max^) were observed for all pH levels tested in the lowest H_2_S addition, corresponding to 450 ppmv. Acetate production and microbial growth subsequently decreased with increasing [TDS]. Overall, we observed that the IC_50_ for homoacetogenic bioproduction (IC_50_^qAc^) in our study lies between 0.72 and 1.86 mM [TDS], 0.34 and 1.27 mM [H_2_S_aq_] and 0.06 and 0.22 mM [HS^‐^] for a pH range of 7‐5 for a mixed microbial community. The average IC_50_^qAc^ [TDS] value corresponds to a [H_2_S_g_] of approximately 6500 ppmv in the gas phase, showing a potentially high resistance of the studied microbial community to sulfide loaded waste gases. The difference in the IC_50_ values between the overall and the maximum acetate production rates implies that a continuous CO_2_ reducing/acetate producing reactor could operate under a sulfide induced ‘inhibited steady state’ (Fotidis *et al.*, 2014). Whilst future applications of a reactor operated at pH 5 with the studied microbial community could lead to lower acetate production rates, it would alleviate the need for extensive addition of chemicals via intensive gas pretreatment steps and pH adjustments. In addition, it needs to be highlighted that the microbial community performing homoacetogenic fermentation could potentially adapt to higher sulfide concentrations over time, which warrants further investigation.

The genera obtained by the 16S rRNA gene amplicon sequencing revealed that this resulted in the formation of a complex microbial system where, instead of solely homoacetogenesis, other metabolic interactions could have taken place, such as sulfur respiration with polysulfides or sulfur as potential electron acceptors. Nevertheless, the main metabolic outcome was still homoacetogenic acetate production. The only known homoacetogen detected in this study, *Acetobacterium*, showed resistance and even enhanced growth in the presence of moderate sulfide concentration (1.26 ± 0.23 mM TDS), compared with the other dominant community members, *Sphingobium* and *Wolinella*. Whilst correlations could be made on sulfide impact on these key community members, definitive statements on key homoacetogens in these communities could not be made. Metatranscriptomic and proteomic studies could allow for identification of the metabolic pathways expressed under sulfide stress and therefore understand whether acetogenesis is conducted under the same pathway. Furthermore, these techniques could shed light upon the differential expression of certain stress factors or mechanisms developed by bacteria to tolerate sulfide stress and help understanding differences in toleration levels, developed among different taxa.

A study of individual homoacetogenic species at different pH and sulfide levels should give important insights on homoacetogenic bioproduction, but would exclude any syntrophic or synergistic interactions that are common in mixed community reactor systems. Future operation of homoacetogenic fermenters will have to consider both the pH and sulfide concentration, as both of them will play, either individually or as combined stresses, a role in the stimulation or inhibition of the bioproduction. Long‐term homoacetogenic fermentation should be investigated to establish to what extent adaptation to higher [TDS] is possible at the various pH levels.

## Experimental procedures

### Enrichment and batch experiments

A mixed microbial community was obtained from the cathode effluent of a working microbial electrosynthesis (MES) reactor, reducing CO_2_ mostly via electrogenerated H_2_ and producing acetate (Patil *et al.*, 2015). To exclude pH interferences on sulfide toxicity, the reactor community was preconditioned at pH 5, pH 6 or pH 7. Cultures were sequentially transferred (10%) four times when a stable acetate production rate was observed. For the sulfide toxicity experiments, the preconditioned pH 5, pH 6 and pH 7 cultures were augmented with increasing sulfide concentrations (0–5 mM [TDS]), by addition of a 100 mM Na_2_S · 9H_2_O stock solution. Initially, the addition of Na_2_S in each serum flask was calculated for a total mass addition of 400 μmol of Na_2_S‐S, equivalent to 10 mM concentration. This resulted in a [TDS] of 5 mM, due to the sulfide dissociation and furthermore to actual concentrations of 3.33 ± 0.34 mM ([TDS]) (average for the three different pH levels), due to reaction of the sulfide with traces of oxygen still remaining in the medium and some of the trace elements. Through the manuscript, the actual [TDS] are reported (detailed in Table S1), taking into account the standard deviation resulting from initial handling of the triplicates. The serum flasks were incubated at 28°C in a horizontal position in order to increase the gas exchange surface. Sampling was conducted over a 240 h period every 48 h, and volatile fatty acids (VFAs), sulfur components, pH, pressure and optical density were measured. Samples for DNA extraction were also taken at the beginning and end of the experiment (240 h). Flow cytometry for total cell counts was conducted in the beginning and at the end of the experiments (*t* = 0 and 240 h).

For both the preconditioning steps and the subsequent batch experiments, 40 ml of media was added to each 250 ml serum flask. The medium was prepared according to Patil *et al. *(2015), excluding cysteine (in order to have only one source of sulfur in the medium) and bicarbonate (leaving CO_2_ as the primary carbon source for acetogenesis) (Table S2). Prior to inoculation, the flasks were sealed with viton rubber stoppers and the headspace flushed with a gas mixture of N_2_:CO_2_, 90:10% through a series of overpressure‐vacuum cycles to create anaerobic conditions. The flasks were subsequently autoclaved (121°C for 20 min). After autoclaving, the headspace was once again exchanged and pressurized to 1.54 ± 0.04 bar with a gas mixture of H_2_ and CO_2_ (H_2_:CO_2_, 70:30%). This two‐step procedure was selected based on safety reasons, to avoid autoclaving a serum flask filled with 70% H_2_ gas.

After the headspace exchange, appropriate pH buffer (1M Tris‐HCl buffer for pH 7 and NaOH adjusted 1M MES for pH 5 and pH 6) and sulfide (from a 100 mM Na_2_S · 9H_2_O stock solution) were added and the serum flasks were left in a horizontal position overnight to equilibrate. After 24 h, 0.4% (v/v) of a vitamin solution [prepared according to Patil *et al. *(2015)] (Table S2) was added. After the equilibration and the addition of the vitamin solution, the actual [TDS] and [H_2_S_aq_] changed, due to binding of initially provided sulfide to metals from the trace element solution (Table S4) or reaction with residual oxygen; therefore, the [TDS] and [H_2_S_aq_] were measured throughout the incubation period in each individual flask. The initial experimental conditions (*t* = 0), e.g. acetate and cell concentrations, pH, [TDS] and [H_2_S_aq_], are provided in Table S1.

### Sampling and analytical methods

Samples were taken from the headspace of the flasks and were analysed for H_2_, O_2_, CO_2_, H_2_S and CH_4_. Samples for sulfur components (sulfide (TDS), sulfite (${\text{SO}}_{3}^{2-}$) and thiosulfate ($S_{2}O_{3}^{2-}$) were immediately prepared for analysis to minimize the potential for oxidation. Samples were taken from experimental flasks with aseptic technique with N_2_/CO_2_‐flushed syringes, and then diluted with treated Milli‐Q water (addition of 50 % v/v NaOH (1:1000) and 37% formaldehyde (1:1000) in Milli‐Q and subsequently flushed with argon). The samples for sulfur components analysis were preserved in freshly prepared sulfide antioxidant buffer (SAOB), according to Keller‐Lehman *et al. *(2006).

The gas‐phase composition was analysed with a Compact GC (Global Analyser Solutions, Breda, The Netherlands), according to Vrieze *et al. *(2016). The total pressure of the serum flasks headspace was measured by using UMS‐Tensiometer (Infield 7) device. The [H_2_Saq] concentrations were calculated by Henry’s law (Equation S1, S1.4), using measured pressure and CGC (compact gas chromatograph) mol % H_2_S. Based on the [H_2_Saq] and the pH measured, the [HS^‐^] was determined with Visual MINTEQ model for acid–base equilibria (Equation S2, S1.3), for pKa_H2Saq/HS‐_ 7.05 (Perrin, 1982) (detailed table (Table S3) with sulfide fractionation in S1.3). The sum of H_2_S_(aq)_ and HS^‐^ was used for the calculation of [TDS], which was cross‐checked with the [TDS] results analysed by IC. The pH was measured with a Metrohm 744 pH meter at room temperature (~ 24°C) (Table S1 and S5), and the OD of the bacterial culture was measured with a UV/Vis spectrophotometer (Isis 9000, Dr Lange, Germany) at 600nm. The OD measurements were normalized to the OD_600_ at t0 (i.e. δOD_600_ = OD_600_ difference between t0 and t each sampling time, e.g. 48 h) at the beginning of the incubation (*t* = 0), and the subsequent measurement (every 48 h) is reported here as δOD_600_ (i.e. the difference between the measured OD_600_ at time *t* = 0 and 240 h)_._ VFA was conducted as previously described, using a 930 Compact Ion Chromatography (IC) Flex (Metrohm, Switzerland) system with inline bicarbonate removal (MCS). Separation was done on a Metrosep organic acid (250/7.8) column at 35°C behind a Metrosep organic acid (4.6) guard column (Gildemyn *et al.*, 2015). Liquid samples prepared for sulfur components were analysed with the same IC with Professional UV/VIS detector Vario and equipped with a Metrosep A Supp 15‐150/4.0 column as described previously (Vaiopoulou *et al.*, 2016).

The sulfide toxicity in the bacterial cells was determined based on overall acetate production rates (q_Ac_), calculated as in Eq. 1. The maximum acetate production rates (q_Ac_^max^), determined between each sampling event, were calculated based on Eq. 2. Partial pressure for each of the gases in the headspace was calculated every sampling event, based on the total pressure of the headspace.

Acetate production rate:

(1) $$q_{\text{Ac}}\;=\;\frac{{\left[{\text{Ac}}^{-}\right]}^{t\;=\;240\;h}-{\left[{\text{Ac}}^{-}\right]}^{t\;=\;0\;h}}{\mathrm{dt}},$$

where q_Ac_ is the acetate production rate in mM h^‐1^, [Ac^−^]*^t^* ^= 240 h^ and [Ac^−^]*^t^* ^= 0 h^ are the acetate concentrations at the end and at the beginning of the experiment, respectively, in mM and dt is the experimental period, in this case 240 h.

Maximum acetate production rate:

(2) $$q_{\text{Ac}}^{\max}=\frac{{\left[{\text{Ac}}^{-}\right]}^{t^{\prime }}-{\left[{\text{Ac}}^{-}\right]}^{t}}{\left(t^{\prime }-t\right)},$$

where q_Ac_^max^ is the maximum acetate production rate in mM h^‐1^ calculated every 48 h of the experimental period, [Ac^−^]*^t^* ^´^ and [Ac^‐^]*^t^* are the acetate concentrations at *t*´=48 + t h and t, respectively, in mM. The IC_50_ values were generated with GraphPad Prism 6 (GraphPad Software, Inc., La Jolla, CA, USA) following a standard log‐dose inhibition curve as in Carlson *et al. *(2015), with 95% confidence intervals (CI). All the values reported are the mean of three biological replicates.

### Total cell counts with flow cytometry (FCM)

In the beginning of the experiment (*t* = 0) and at experimental end‐points (*t* = 240 h), samples were taken from each serum flask following the aforementioned sampling procedure for total cell counts with FCM. The samples were appropriately diluted with PBS buffer and afterwards stained with SYBR Green I (SG), suitable for a total cell count. The SYBR Green I (10 000× concentrate in DMSO; Invitrogen; Thermo Fischer Scientific, Merelbeke, Belgium) stain was diluted 100 times in 0.22‐µm‐filtered DMSO (IC Millex; Merck Chemicals, Overijse, Belgium). The samples were stained with 10 µl ml^−1^ staining solution according to Prest *et al. *(2013) and incubated for 13 min at 37°C.

All measurements were conducted with a FACSVerse cytometer (BD Biosciences, Erembodegem, Belgium). The instrument was calibrated with the CS&T calibration beads (BD Biosciences) daily. The blue laser (488 nm) was used for the excitation of the stains. The optical filters used were 527 nm with a bandpass of 32 nm for the green fluorescence and 700 nm with a bandpass of 54 nm for the red fluorescence. A minimum of 10 000 cells per sample were measured to allow accurate quantification. The data of each sample were denoised from (in)organic noise by a filtering approach using the flowCorepackage (v1.38.1) in R (v3.3.2). The bacterial cell population was extracted by a manual gate applied on the primary fluorescence emission channels.

### 16S rRNA Gene amplicon sequencing

Samples for DNA extraction were taken at *t* = 0 and *t* = 240 h from the triplicate serum flasks representing of sets 1, 6 and 8 (Table S1), representing averaged (for the pH 7, pH 6 and pH 5) total dissolved sulfide concentrations of 0.06 ± 0.01, 1.26 ± 0.23 and 3.33 ± 0.34 mM [TDS]. Throughout the manuscript, these concentrations will be reported as 0.06, 1.26 and 3.33 mM [TDS]. The samples were pelleted by centrifugation for 10 min at 10 000* g*. Pellets were stored at −20°C till further processing. DNA was extracted according to Vilchez‐Vargas *et al. *(2013) (Vilchez‐Vargas *et al.*, 2013). DNA quality was evaluated on a 1% (w/v) agarose gel. 16S rRNA gene amplicon sequencing analysis was performed as described before (Vrieze *et al.*, 2016; Domingos *et al.*, 2017). Near full‐length 16S rRNA gene sequencing was performed using the Sanger method. Information on the DNA extraction methods, gene amplicon sequencing analysis, clone library methods and data processing details can be found in Supporting information.

All statistical sequence analysis was performed in R (v3.3.2). The reads received from 16S rRNA gene amplicon sequencing were imported in R. OTUs with no more than one read in every sample (singletons) were removed (McMurdie and Holmes, 2014). The estimated absolute abundances (EAAs) of the different genera were calculated by projecting the relative abundances, obtained by sequencing, to the cell numbers obtained by FCM (Props *et al.*, 2016). The graphs representing the 15 most relative or absolute abundant genera were generated using the phyloseq package 7 in R (v3.3.2). Non‐metric multidimensional scaling (NMDS) plots of relative abundances were prepared based on the Jaccard distance to visualize the effect of pH and [TDS] on the β‐diversity. The confidence ellipses were computed using the function ‘https://github.com/tidyverse/ggplot2/blob/master/R/stat-ellipse.R' in R (v 3.3.2) with confidence level 0.95.

### Accession number(s)

The flow cytometry data (.fcs format) have been submitted to the FlowRepository archive under repository ID FR‐FCM‐ZYX5. The sequences of the 16S rRNA gene have been submitted to the NCBI Sequence Read Archive (SRA) under accession number SRP157026.

## Conflict of interest

None declared.

## Supporting information


**Fig. S1.** Initial inoculation at different pH values. Every inoculation lasted for ~7 days and on the figure, 4 consecutive transfers are displayed.
**Fig. S2. **Total cell counts for the different incubation experiments obtained with flow cytometry (FCM) at *t* = 0 h and *t* = 240 h. In the plot the average TDS concentrations of the different incubations are presented, at pH 7 (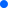), 6 (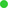) and 5 (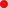), respectively. Data are averages of 3 incubations, error bars represent standard deviations of biological triplicates.
**Fig. S3. **Estimated Absolute Abundances (EEA) in (cells ml^‐1^) of the 15 most abundant OTUs, calculated as relative abundances normalised for the flow cytometric counts at t0, after culture preconditioning (4 transfers over 28 days) at pH 7, 6 and 5, respectively.
**Fig. S4. **Time course graph for acetate production (mM) at pH 5 and 0.06, 1.26 and 3.33 mM TDS. Example representative of all incubations at different pH levels and TDS concentrations. Data are averages of 3 incubations, error bars represent standard deviations of biological triplicates.
**Fig. S5. **Total acetate production rate (qAc) (mM h‐1) as a function of: (A) initial total dissolved sulfide concentration ([TDS]) (mM), (B) initial hydrogen sulfide dissolved ([H2Saq]) (mM) and (C) initial bisulfide concentration ([HS‐]) (mM) at pH 7 (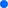), 6 (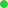) and 5 (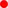). Data are averages of 3 incubations, error bars represent standard deviations of biological triplicates.
**Fig. S6. **Maximum acetate production rate (mM h‐1), calculated in a 48 h basis, plotted against: (A) initial total dissolved sulfide concentration ([TDS]) (mM), (B) initial dissolved hydrogen sulfide concentration ([H2Saq]) (mM) and (C) initial bisulfide concentration ([HS‐]) (mM) at Ph 7 (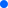), 6 (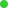) and 5 (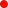). Data are averages of 3 incubations, error bars represent standard deviations of biological triplicates.
**Fig. S7. **Relative Abundances (%) of the 15 most abundant OTUs, in three biological replicates (2 biological replicates at pH 6 and 1.26 mM [TDS]) at *t* = 240 h.
**Table S1. **Initial conditions in flask experiments. Three pH values (7, 6 and 5) were tested with 8 initial sulfide concentrations in triplicate. Initial (*t* = 0) total dissolved sulfide concentration ([TDS], mM), measured pH values, H_2_Saq concentration ([H_2_Saq], mM), acetate concentration ([Ac‐], mM) and cell density ([Cells], Cells ml^‐1^) after inoculation are presented.
**Table S2. **Composition of modified homoacetogenic medium adapted from Patil et al. (2015) (Patil et al. 2015).
**Table S3. **Sulfide fractionation based on acid‐base equilibria for a pH = 4.5– 7.5, derived by Visual MINTEQ.
**Table S4. **Metal availability with increasing sulfide concentrations at the end of the experimental cycle (*t* = 240 h) conducted at pH 7, analysed with ICP‐MS, as previously described (Folens et al. 2018).
**Table S5. **Maximum values obtained during the batch experiments, for the 3 different runs (pH 7, 6 and 5). Total acetate production rate (qAc, mM h^‐1^), maximum acetate production rate (qAcmax, mM h^‐1^), maximum delta optical density measured at 600 nm (δOD600max) and cell density ([Cells], Cells mL^‐1^), measured at the end of each batch experiment (*t* = 240 h) are presented.
