# Supplementary material for: Homoacetogenesis and microbial community composition are shaped by pH and total sulfide concentration
Source: Microb Biotechnol. 2020 Mar 3;13(4):1026–38. doi: 10.1111/1751-7915.13546 (PMC7264883; doi:10.1111/1751-7915.13546)
Supplement: Supplementary file 1 — Fig. S1. Initial inoculation at different pH values. Every inoculation lasted for ~7 days and on the figure, 4 consecutive transfers are displayed. Fig. S2. Total cell counts for the different incubation experiments obtained with flow cytometry (FCM) at t = 0 h and t = 240 h. In the plot the average TDS concentrations of the different incubations are presented, at pH 7 (), 6 () and 5 (), respectively. Data are averages of 3 incubations, error bars represent standard deviations of biological triplicates. Fig. S3. Estimated Absolute Abundances (EEA) in (cells ml‐1) of the 15 most abundant OTUs, calculated as relative abundances normalised for the flow cytometric counts at t0, after culture preconditioning (4 transfers over 28 days) at pH 7, 6 and 5, respectively. Fig. S4. Time course graph for acetate production (mM) at pH 5 and 0.06, 1.26 and 3.33 mM TDS. Example representative of all incubations at different pH levels and TDS concentrations. Data are averages of 3 incubations, error bars represent standard deviations of biological triplicates. Fig. S5. Total acetate production rate (qAc) (mM h‐1) as a function of: (A) initial total dissolved sulfide concentration ([TDS]) (mM), (B) initial hydrogen sulfide dissolved ([H2Saq]) (mM) and (C) initial bisulfide concentration ([HS‐]) (mM) at pH 7 (), 6 () and 5 (). Data are averages of 3 incubations, error bars represent standard deviations of biological triplicates. Fig. S6. Maximum acetate production rate (mM h‐1), calculated in a 48 h basis, plotted against: (A) initial total dissolved sulfide concentration ([TDS]) (mM), (B) initial dissolved hydrogen sulfide concentration ([H2Saq]) (mM) and (C) initial bisulfide concentration ([HS‐]) (mM) at Ph 7 (), 6 () and 5 (). Data are averages of 3 incubations, error bars represent standard deviations of biological triplicates. Fig. S7. Relative Abundances (%) of the 15 most abundant OTUs, in three biological replicates (2 biological replicates at pH 6 and 1.26 mM [TDS]) at t = 2 [file MBT2-13-1026-s001.docx]

Supplementary Material for

**Homoacetogenesis and microbial community composition are shaped by pH and total sulfide concentration**

**Eleftheria Ntagia, Ioanna Chatzigiannidou, Adam J. Williamson, Jan B. A. Arends, Korneel Rabaey***

Center for Microbial Ecology and Technology (CMET), Ghent University,

Coupure Links 653, 9000 Ghent 9, Belgium

*Corresponding author (korneel.rabaey@ugent.be)

**1 Experimental procedures**

**1.1 16S rRNA Gene amplicon sequencing**. In brief, DNA extraction was performed by means of bead beating with a PowerLyzer instrument (Qiagen, Venlo, Netherlands) and phenol/chloroform extraction. The 16S rRNA gene V3-V4 hypervariable regions were amplified by PCR using primers 341F (5’-CCT ACG GGN GGC WGC AG -3’) and 785Rmod (5’-GAC TAC HVG GGT ATC TAA KCC-3’). The reverse primer was adapted from Klindworth et al. (Klindworth et al. 2013), to increase coverage. PCR was performed using Taq DNA Polymerase with the Fermentas PCR Kit according to the manufacturers’ specifications (ThermoFisher Scientific, Waltham, MA, USA). The obtained PCR product was ran on a 2% agarose gel for 30 minutes at 100V. 10µl of the original genomic DNA extract was send out to LGC Genomics GmbH (Berlin, Germany) for library preparation and sequencing on an Illumina Miseq platform with v3 chemistry with the primers mentioned above. Read assembly and cleanup was largely derived from the MiSeq SOP described by the Schloss lab (Schloss et al. 2011; Kozich et al. 2013). In brief, mothur (v.1.39.5) was used to assemble reads into contigs, perform alignment-based quality filtering (alignment to the mothur-reconstructed SILVA SEED alignment, v. 123), remove chimeras, assign taxonomy using a naïve Bayesian classifier (Wang et al. 2007) and and SILVA NR v128 and cluster contigs into OTUs at 97% sequence similarity. All sequences that were classified as Eukaryota, Archaea, Chloroplasts and Mitochondria were removed. Also, if sequences could not be classified at all (even at (super)Kingdom level) they were removed. For each OTU representative sequences were picked as the most abundant sequence within that OTU.

**1.2 Clone library**. PCR was performed using a recombinant Taq DNA Polymerase kit, Fermentas (Thermo Fischer Scientific, Waltham, MA, USA), in 25µl reactions containing: 2.5 µl 10x Taq buffer (+KCl – MgCl_2_), 0.5 µl of 10mM dNTP, 2 µl of 25 mM MgCl_2_, 2.5 µl of 10µM Primer 27f AGAGTTTGATCMTGGCTCAG, 2.5 µl of 10µM Primer 1492r TACGGYTACCTTGTTACGACTT, 0.125 µl of 5 U/µL Taq polymerase, 0.065 µl of 20mg/ml BSA (Roche Holding AG, Basel, Switzerland), 14.81 µl PCR-water and 1 µl Sample. Amplification was ran including initial denaturation for 7 min at 95°C, followed by 32 cycles of 1 min denaturation at 95°C, 1 min anneal at 55°C and 2 min extension at 72°C. A final elongation step was included at 72°C for 10 min. The product was ran for 30 min at 100V on a 2% agarose gel and was visualized using a UV transilluminator. The fragment was purified using the innuPREP PCRpure Kit (Analytik Jena, Jena, Germany) and the concentration was measured with a DS-11 Microvolume Spectrophotometer (DeNovix, Wilmington, DE,  USA). The purified fragment was cloned into a pCR™2.1-TOPO® vector and subsequently transformed into One Shot® DH5α™-TOP10 cells using the TOPO® TA Cloning® kit (Thermo Fisher Scientific, Waltham, MA, USA). Following blue-white screening, 16 white colonies were picked and used in colony-PCR using the above mentioned protocol and primers M13F GTAAAACGACGGCCAG and M13R CAGGAAACAGCTATGAC. The PCR was checked on a 2% agarose gel, the 16 positive products were purified with the innuPREP PCRpure Kit and their concentrations measured with a DS-11 Microvolume Spectrophotometer (DeNovix, Wilmington, DE,  USA). Subsequently, 4 µl from a 5 µM stock of one of the M13 primers was added to 10 µl of 40 ng/µl of PCR product, for both forward and reverse sequencing reactions. The reactions were send out to LGC Genomics (LGC Genomics GmbH, Berlin, Germany). The resulting sequences were blasted using NCBI’s BLAST ( https://blast.ncbi.nlm.nih.gov/Blast.cgi).

**1.3 Experimental procedure and calculations**

The [H_2_Saq] concentrations were calculated using measured pressure and CGC (Compact Gas Chromatograph) mol % H_2_S, according to the following equation:

$\left[ {H_{2}S}_{aq} \right]=K_{H2S}*P_{H2S}= K_{H2S}*P_{tot}*mol\%H_{2}S$ (Equation S1)

, where K_H2S_ = 10^-3^ mol m^-3^ Pa^-1^ (Sander 2015)

${pKa}_{H2S/ HS-}=log\frac{[{H_{2}S}_{aq}]}{\left[ {HS}^{-} \right]\times[H^{+}]}$ (Equation S2)

**Table S1** – Initial conditions in flask experiments. Three pH values (7, 6 and 5) were tested with 8 initial sulfide concentrations in triplicate. Initial (t=0) total dissolved sulfide concentration ([TDS], mM), measured pH values, H_2_S_aq_ concentration ([H_2_S_aq_], mM), acetate concentration ([Ac^-^], mM) and cell density ([Cells], Cells mL^-1^) after inoculation are presented.

| **Run** | **Set** | **[TDS] (mM)** | **pH** | **[H_2_S_aq_] (mM)** | **[Ac^-^] (mM)** | **[Cells] (10^7^ Cells) mL^-1^)** |
| --- | --- | --- | --- | --- | --- | --- |
|  | Average | n.a. | 6.81 ± 0.12 | n.a. | 3.32 ± 0.23 | 7.36 ± 0.89 |
| **pH 7** | 1 | 0.06 ± 0.01 | 6.80 ± 0.09 | 0.04 ± 0.01 | 3.48 ± 0.11 | 7.63 ± 0.56 |
|  | 2 | 0.18 ± 0.04 | 6.74 ± 0.05 | 0.12 ± 0.02 | 3.41 ± 0.29 | 7.35 ± 0.95 |
|  | 3 | 0.31 ± 0.04 | 6.73 ± 0.04 | 0.20 ± 0.03 | 3.39 ± 0.09 | 7.05 ± 1.04 |
|  | 4 | 0.58 ± 0.06 | 6.78 ± 0.07 | 0.37 ± 0.04 | 3.27 ± 0.36 | 7.53 ± 0.96 |
|  | 5 | 0.93 ± 0.05 | 6.67 ± 0.08 | 0.63 ± 0.01 | 3.30 ± 0.23 | 6.94 ± 1.32 |
|  | 6 | 1.56 ± 0.25 | 6.89 ± 0.06 | 0.79 ± 0.06 | 3.35 ± 0.09 | 8.05 ± 0.05 |
|  | 7 | 1.73 ± 0.24 | 6.86 ± 0.09 | 0.96 ± 0.05 | 3.15 ± 0.15 | 7.27 ± 0.65 |
|  | 8 | 3.79 ± 0.34 | 6.99 ± 0.06 | 1.96 ± 0.09 | 3.21 ± 0.17 | 7.05 ± 0.25 |
|  | Average | n.a. | 6.06 ± 0.16 | n.a. | 1.77 ± 0.25 | 6.13 ± 0.79 |
| **pH 6** | 1 | 0.06 ± 0.01 | 5.91 ± 0.04 | 0.06 ± 0.01 | 2.04 ± 0.42 | 6.07 ± 0.77 |
|  | 2 | 0.13 ± 0.03 | 5.94 ± 0.01 | 0.12 ± 0.03 | 1.85 ± 0.13 | 5.72 ± 0.68 |
|  | 3 | 0.27 ± 0.05 | 5.95 ± 0.00 | 0.24 ± 0.05 | 1.90 ± 0.01 | 6.46 ± 0.71 |
|  | 4 | 0.51 ± 0.07 | 6.02 ± 0.04 | 0.46 ± 0.06 | 1.73 ± 0.12 | 5.60 ± 0.90 |
|  | 5 | 0.85 ± 0.04 | 6.05 ± 0.04 | 0.76 ± 0.04 | 1.70 ± 0.18 | 6.20 ± 0.52 |
|  | 6 | 0.99 ± 0.12 | 6.09 ± 0.02 | 0.88 ± 0.11 | 1.63 ± 0.27 | 5.81 ± 0.45 |
|  | 7 | 1.18 ± 0.10 | 6.15 ± 0.04 | 1.04 ± 0.10 | 1.69 ± 0.18 | 6.38 ± 0.76 |
|  | 8 | 3.00 ± 0.21 | 6.41 ± 0.04 | 2.37 ± 0.21 | 1.64 ± 0.16 | 6.83 ± 0.58 |
|  | Average | n.a. | 5.58 ± 0.28 | n.a. | 1.35 ± 0.10 | 6.13 ± 0.49 |
| **pH 5** | 1 | 0.04 ± 0.00 | 5.23 ± 0.03 | 0.04 ± 0.00 | 1.43 ± 0.07 | 6.22 ± 0.32 |
|  | 2 | 0.20 ± 0.02 | 5.29 ± 0.02 | 0.20 ± 0.02 | 1.46 ± 0.04 | 6.48 ± 0.19 |
|  | 3 | 0.30 ± 0.02 | 5.39 ± 0.01 | 0.29 ± 0.02 | 1.43 ± 0.03 | 6.64 ± 0.37 |
|  | 4 | 0.63 ± 0.03 | 5.52 ± 0.03 | 0.61 ± 0.03 | 1.37 ± 0.05 | 5.97 ± 0.17 |
|  | 5 | 0.78 ± 0.02 | 5.58 ± 0.02 | 0.75 ± 0.02 | 1.37 ± 0.05 | 6.10 ± 0.37 |
|  | 6 | 1.24 ± 0.14 | 5.72 ± 0.03 | 1.18 ± 0.13 | 1.35 ± 0.05 | 5.77 ± 0.41 |
|  | 7 | 1.45 ± 0.06 | 5.80 ± 0.03 | 1.37 ± 0.06 | 1.26 ± 0.05 | 6.23 ± 0.32 |
|  | 8 | 3.19 ± 0.07 | 6.13 ± 0.05 | 2.81 ± 0.06 | 1.17 ± 0.06 | 5.60 ± 0.63 |

**Table S2** – Composition of modified homoacetogenic medium adapted from Patil et al. (2015) (Patil et al. 2015)

| K_2_HPO_4_ | 0.2 g L^-1^ |
| --- | --- |
| NH_4_Cl | 0.25 g L^-1^ |
| KCl | 0.5 g L^-1^ |
| CaCl_2_.2H_2_O | 0.15 g L^-1^ |
| MgCl_2_.6H_2_O | 0.6 g L^-1^ |
| NaCl | 1.2 g L^-1^ |
| Tryptone | 0.2 g L^-1^ |
| Yeast extract | 0.5 g L^-1^ |
| Addition in 1 L of medium |  |
| Trace metal solution* | 1 mL |
| Vitamin solution** | 2.5 mL |
| Tungstate-selenium solution*** | 0.1 mL |

| ***Composition of trace metal solution** (g L^-1^) |  | |
| --- | --- | --- |
| Nitrilotriacetic acid (dissolve with KOH; pH 6.5) | 1.5 | |
| Mg_2_Cl_2_.6H_2_O | 3.0 | |
| MnCl_2_.2H_2_O | 0.5 | |
| NaCl | 1 | |
| FeCl_2_ | 0.1 | |
| CoCl_2_ | 0.1 | |
| CaCl_2_.2H_2_O | 0.1 | |
| ZnCl_2_ | 0.1 | |
| CuCl_2_ | 0.01 | |
| AlCl_3_.6H_2_O | 0.01 | |
| H_3_BO_3_ | 0.01 | |
| Na_2_MoO_4_.2H_2_O | 0.01 | |
| ****Composition of vitamin solution** (mg L^-1^) |  |  |
| Sodium ascorbate | 10 | |
| Biotin | 4 |  |
| Folic acid | 4 |  |
| Pyridoxine hydrochoride | 20 |  |
| Thiamine hydrocloride | 10 |  |
| Riboflavin | 10 |  |
| Nicotinic acid | 10 |  |
| DL-calcium pantothenate | 10 |  |
| Vitamin B12 | 0.2 |  |
| p-aminobenzoic acid | 10 |  |
| Lipoic(thioctic) acid | 10 |  |
| Myo-inositol | 10 |  |
| Choline chloride | 10 |  |
| Niacinamide | 10 |  |
| Pyridoxal hydrochloride | 10 |  |
| *****Composition of tunstate- selenium solution**  0.1mM Na_2_WO_4_ + 0.1mM Na_2_SeO_3_ in 20mM NaOH |  |  |
|  |  |  |

**Table S3 –** Sulfide fractionation based on acid-base equilibria for a pH = 4.5 – 7.5, derived by Visual MINTEQ

| pH | HS^-^ % | H_2_S (aq) % |
| --- | --- | --- |
| 4.5 | 0.335 | 99.665 |
| 4.6 | 0.422 | 99.578 |
| 4.7 | 0.532 | 99.468 |
| 4.8 | 0.670 | 99.330 |
| 4.9 | 0.844 | 99.156 |
| 5.0 | 1.064 | 98.936 |
| 5.1 | 1.341 | 98.659 |
| 5.2 | 1.690 | 98.310 |
| 5.3 | 2.128 | 97.872 |
| 5.4 | 2.364 | 97.636 |
| 5.5 | 2.960 | 97.040 |
| 5.6 | 3.703 | 96.297 |
| 5.7 | 4.623 | 95.377 |
| 5.8 | 5.758 | 94.242 |
| 5.9 | 7.153 | 92.847 |
| 6.0 | 8.856 | 91.144 |
| 6.1 | 10.917 | 89.083 |
| 6.2 | 13.389 | 86.611 |
| 6.3 | 16.320 | 83.680 |
| 6.4 | 19.748 | 80.252 |
| 6.5 | 23.695 | 76.305 |
| 6.6 | 28.155 | 71.845 |
| 6.7 | 33.092 | 66.908 |
| 6.8 | 38.430 | 61.570 |
| 6.9 | 44.062 | 55.938 |
| 7.0 | 49.848 | 50.152 |
| 7.1 | 55.634 | 44.366 |
| 7.2 | 61.268 | 38.732 |
| 7.3 | 66.611 | 33.389 |
| 7.4 | 71.555 | 28.445 |
| 7.5 | 76.027 | 23.973 |

**2 Results**

**Table S4** – Metal availability with increasing sulfide concentrations at the end of the experimental cycle (t = 240 h) conducted at pH 7, analysed with ICP-MS, as previously described (Folens et al. 2018).

| **samples** | **[TDS] (mM)** | **Fe (µg L^-1^)** | **Mn (µg L^-1^)** | **Mg (mg L^-1^)** |
| --- | --- | --- | --- | --- |
| inoculated | 0.06 | 634 | 168 | 74.7 |
| inoculated | 1.26 | 254 | 159 | 69.6 |
| inoculated | 3.33 | 241 | 157 | 64.8 |
| control | 0.06 | 854 | 209 | 80.3 |
| control | 1.26 | 424 | 208 | 78.1 |
| control | 3.33 | 384 | 187 | 72.3 |

**Table S5** - Maximum values obtained during the batch experiments, for the 3 different runs (pH 7, 6 and 5). Total acetate production rate (q_Ac_, mM h^-1^), maximum acetate production rate (q_Ac_^max^, mM h^-1^), maximum delta optical density measured at 600 nm (δOD_600_^max^) and cell density ([Cells], Cells mL^-1^), measured at the end of each batch experiment (t=240h) are presented.

| **Run** | **Set** | **q_Ac_**  **(mM h^-1^)** | **q_Ac_^max^**  **(mM h^-1^)** | **δOD_600_^max^**  **(-)** | **[Cells] (10^7^ Cells mL^-1^)** | **pH** |
| --- | --- | --- | --- | --- | --- | --- |
|  | Average | n.a. | n.a. | n.a. | n.a. | 6.56 ± 0.34 |
| **pH 7** | 1 | 0.12 ± 0.03 | 0.21 ± 0.08 | 0.71 ± 0.14 | 95.2 ± 6.77 | 6.03 ± 0.45 |
|  | 2 | 0.11 ± 0.01 | 0.15 ± 0.07 | 0.57 ± 0.02 | 91.7 ± 24.5 | 6.31 ± 0.17 |
|  | 3 | 0.07 ± 0.01 | 0.09 ± 0.04 | 0.57 ± 0.11 | 95.1 ± 11.7 | 6.56 ± 0.05 |
|  | 4 | 0.09 ± 0.01 | 0.10 ± 0.00 | 0.42 ± 0.05 | 65.4 ± 9.96 | 6.49 ± 0.03 |
|  | 5 | 0.09 ± 0.01 | 0.11 ± 0.01 | 0.33 ± 0.04 | 48.1 ± 4.67 | 6.47 ± 0.10 |
|  | 6 | 0.04 ± 0.01 | 0.05 ± 0.02 | 0.24 ± 0.02 | 51.9 ± 3.28 | 6.80 ± 0.10 |
|  | 7 | 0.03 ± 0.02 | 0.04 ± 0.03 | 0.20 ± 0.07 | 44.2 ± 1.79 | 6.83 ± 0.05 |
|  | 8 | 0.01 ± 0.01 | 0.01 ± 0.02 | 0.15 ± 0.08 | 21.6 ± 3.62 | 6.99 ± 0.03 |
|  | Average | n.a. | n.a. | n.a. | n.a. | 5.47 ± 0.38 |
| **pH 6** | 1 | 0.09 ± 0.02 | 0.20 ± 0.02 | 0.74 ± 0.12 | 72. ± 8.53 | 5.09 ± 0.11 |
|  | 2 | 0.12 ± 0.00 | 0.24 ± 0.03 | 0.89 ± 0.06 | 87.4 ± 11.9 | 4.99 ± 0.03 |
|  | 3 | 0.10 ± 0.00 | 0.19 ± 0.02 | 0.78 ± 0.10 | 84.5 ± 16.0 | 5.21 ± 0.03 |
|  | 4 | 0.09 ± 0.01 | 0.16 ± 0.04 | 0.67 ± 0.09 | 88.9 ± 22.2 | 5.44 ± 0.07 |
|  | 5 | 0.08 ± 0.00 | 0.14 ± 0.01 | 0.51 ± 0.05 | 90.9 ± 0.00 | 5.55 ± 0.03 |
|  | 6 | 0.09 ± 0.01 | 0.16 ± 0.07 | 0.49 ± 0.12 | 62.9 ± 14.5 | 5.53 ± 0.10 |
|  | 7 | 0.07 ± 0.02 | 0.14 ± 0.03 | 0.54 ± 0.15 | 56.1 ± 10.6 | 5.71 ± 0.10 |
|  | 8 | 0.02 ± 0.00 | 0.02 ± 0.01 | 0.08 ± 0.01 | 21.6 ± 2.03 | 6.27 ± 0.01 |
|  | Average | n.a. | n.a. | n.a. | n.a. | 5.21 ± 0.45 |
| **pH 5** | 1 | 0.04 ± 0.00 | 0.12 ± 0.02 | 0.71 ± 0.10 | 100 ± 29.5 | 4.80 ± 0.07 |
|  | 2 | 0.05 ± 0.00 | 0.10 ± 0.02 | 0.71 ± 0.12 | 92.6 ± 22.8 | 4.79 ± 0.04 |
|  | 3 | 0.05 ± 0.00 | 0.10 ± 0.01 | 0.75 ± 0.10 | 94.5 ± 11.5 | 4.84 ± 0.04 |
|  | 4 | 0.06 ± 0.00 | 0.11 ± 0.01 | 0.66 ± 0.04 | 96.1 ± 19.2 | 4.96 ± 0.01 |
|  | 5 | 0.05 ± 0.01 | 0.09 ± 0.03 | 0.48 ± 0.04 | 76.0 ± 23.0 | 5.13 ± 0.15 |
|  | 6 | 0.04 ± 0.00 | 0.09 ± 0.01 | 0.33 ± 0.04 | 72.5 ± 6.85 | 5.44 ± 0.03 |
|  | 7 | 0.03 ± 0.00 | 0.05 ± 0.01 | 0.22 ± 0.08 | 39.4 ± 22.6 | 5.67 ± 0.04 |
|  | 8 | 0.01 ± 0.00 | 0.03 ± 0.02 | 0.09 ± 0.02 | 12.9 ± 0.54 | 6.09 ± 0.01 |


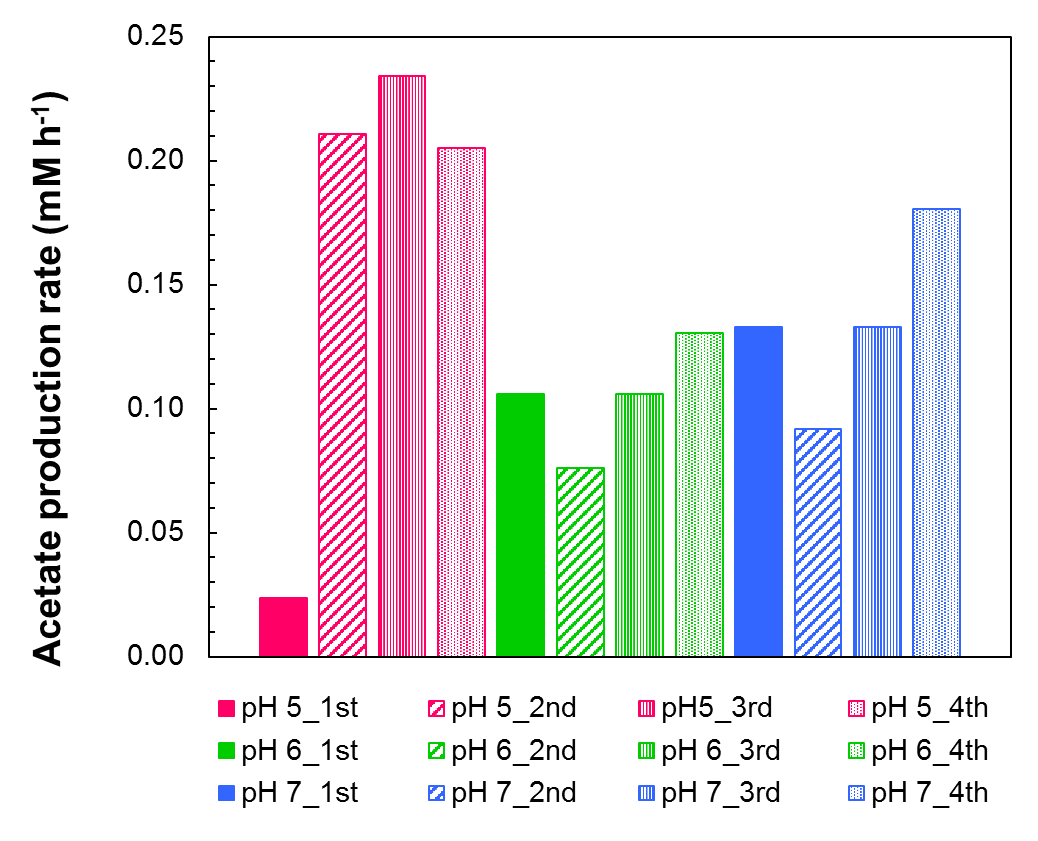


**Fig. S1** - Initial inoculation at different pH values. Every inoculation lasted for ~7 days and on the figure, 4 consecutive transfers are displayed.


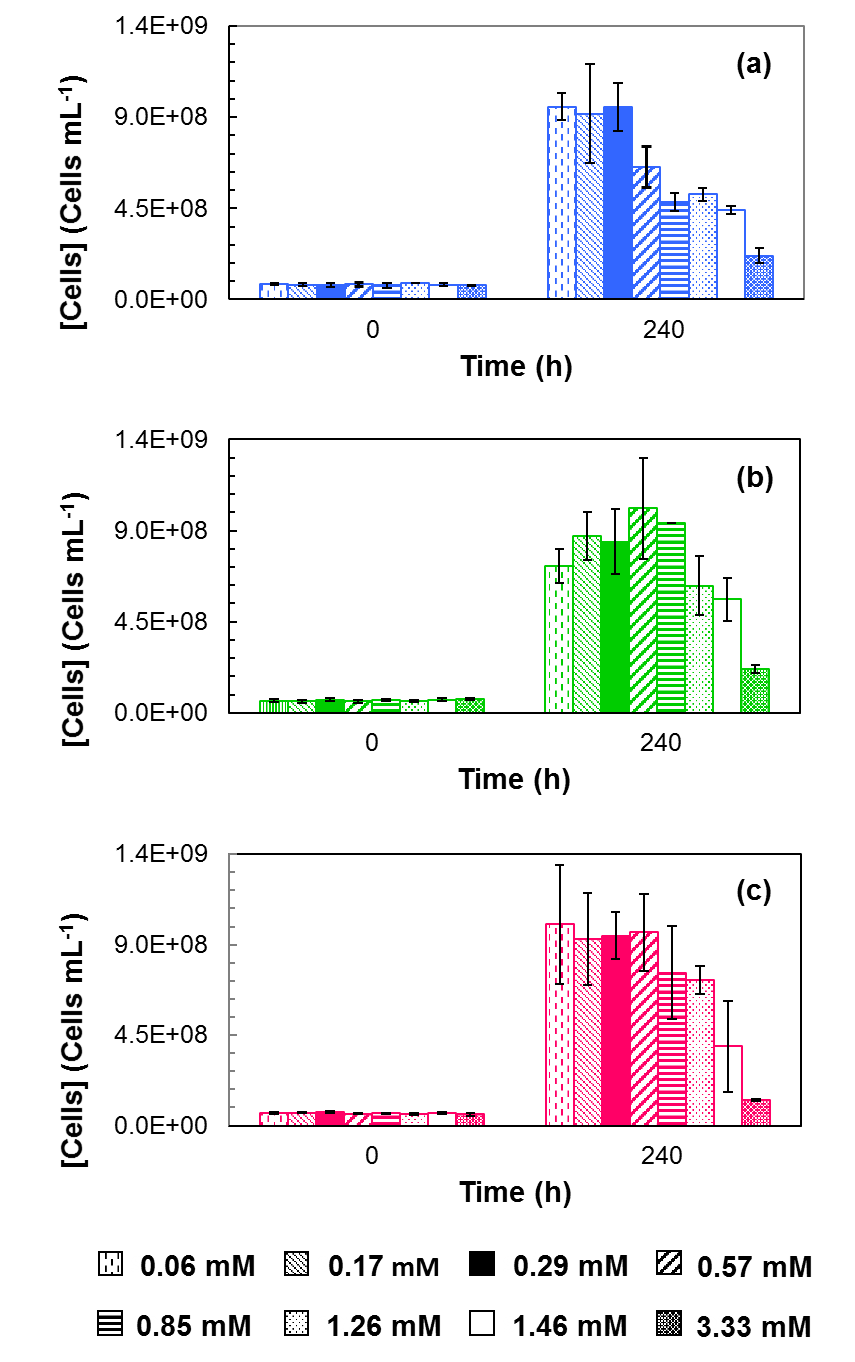


**Fig. S2** - Total cell counts for the different incubation experiments obtained with flow cytometry (FCM) at t=0 h and t=240 h. In the plot the average TDS concentrations of the different incubations are presented, at pH 7 ( ), 6 ( ) and 5 ( ), respectively. Data are averages of 3 incubations, error bars represent standard deviations of biological triplicates.

*
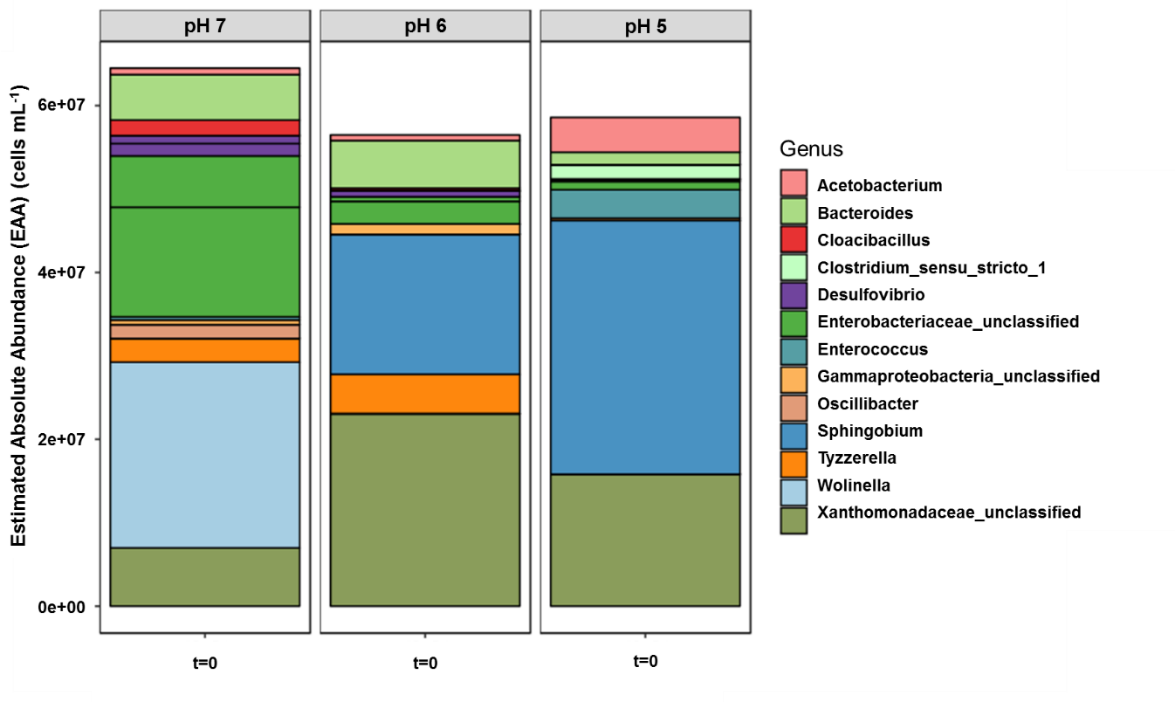
*

**Fig. S3** - Estimated Absolute Abundances (EEA) in (cells mL^-1^) of the 15 most abundant OTUs, calculated as relative abundances normalised for the flow cytometric counts at t0, after culture preconditioning (4 transfers over 28 days) at pH 7, 6 and 5, respectively.


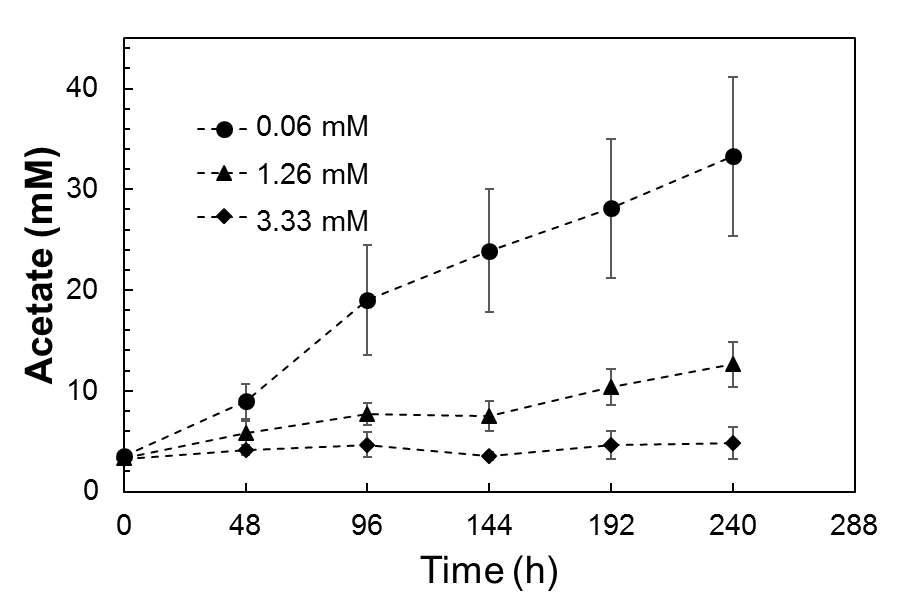


**Fig. S4** – Time course graph for acetate production (mM) at pH 5 and 0.06, 1.26 and 3.33 mM TDS. Example representative of all incubations at different pH levels and TDS concentrations. Data are averages of 3 incubations, error bars represent standard deviations of biological triplicates.


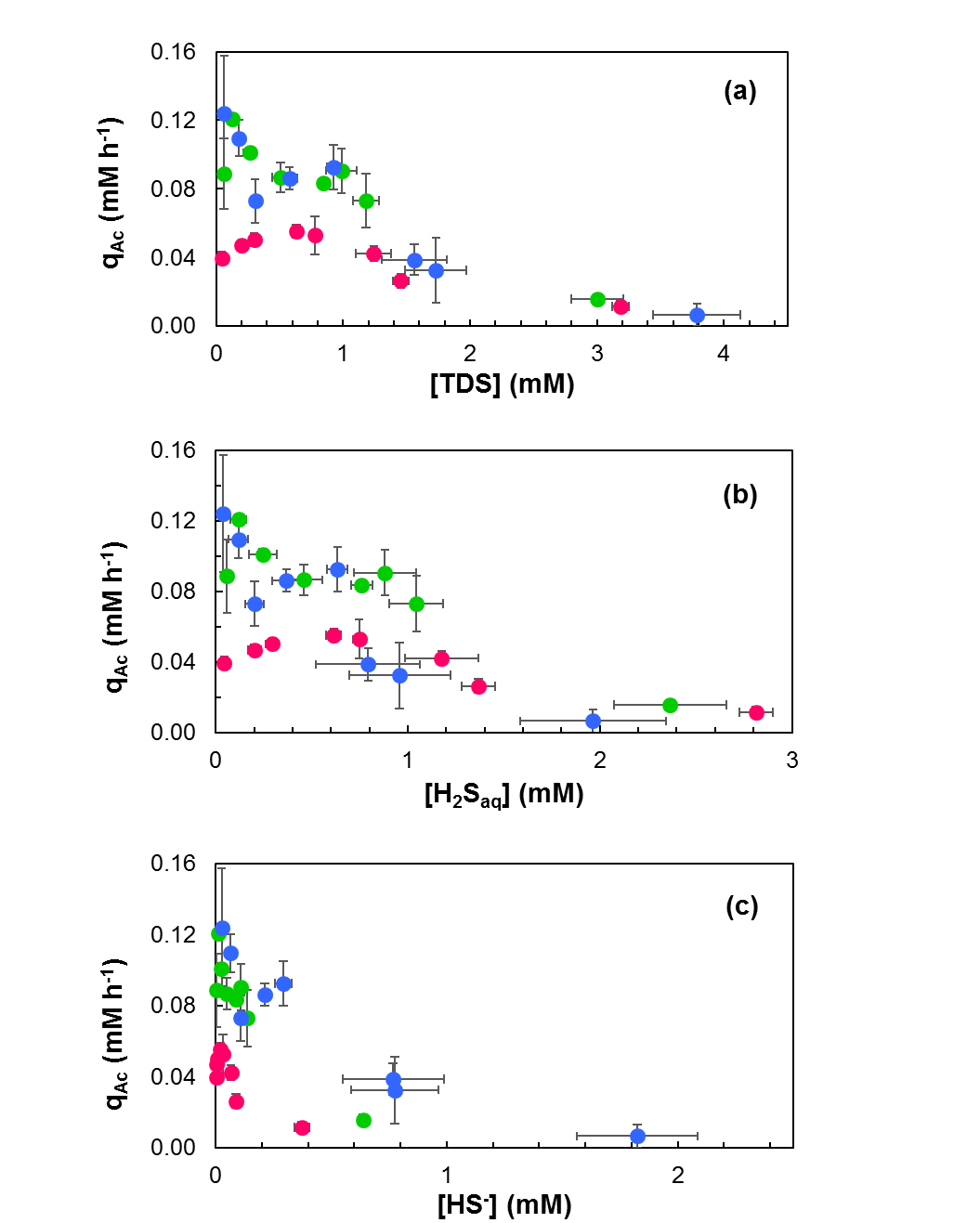


**Fig. S5** - Total acetate production rate (q_Ac_) (mM h^-1^) as a function of: (a) initial total dissolved sulfide concentration ([TDS]) (mM), (b) initial hydrogen sulfide dissolved ([H_2_S_aq_]) (mM) and (c) initial bisulfide concentration ([HS^-^]) (mM) at pH 7 ( ), 6 ( ) and 5 ( ). Data are averages of 3 incubations, error bars represent standard deviations of biological triplicates.


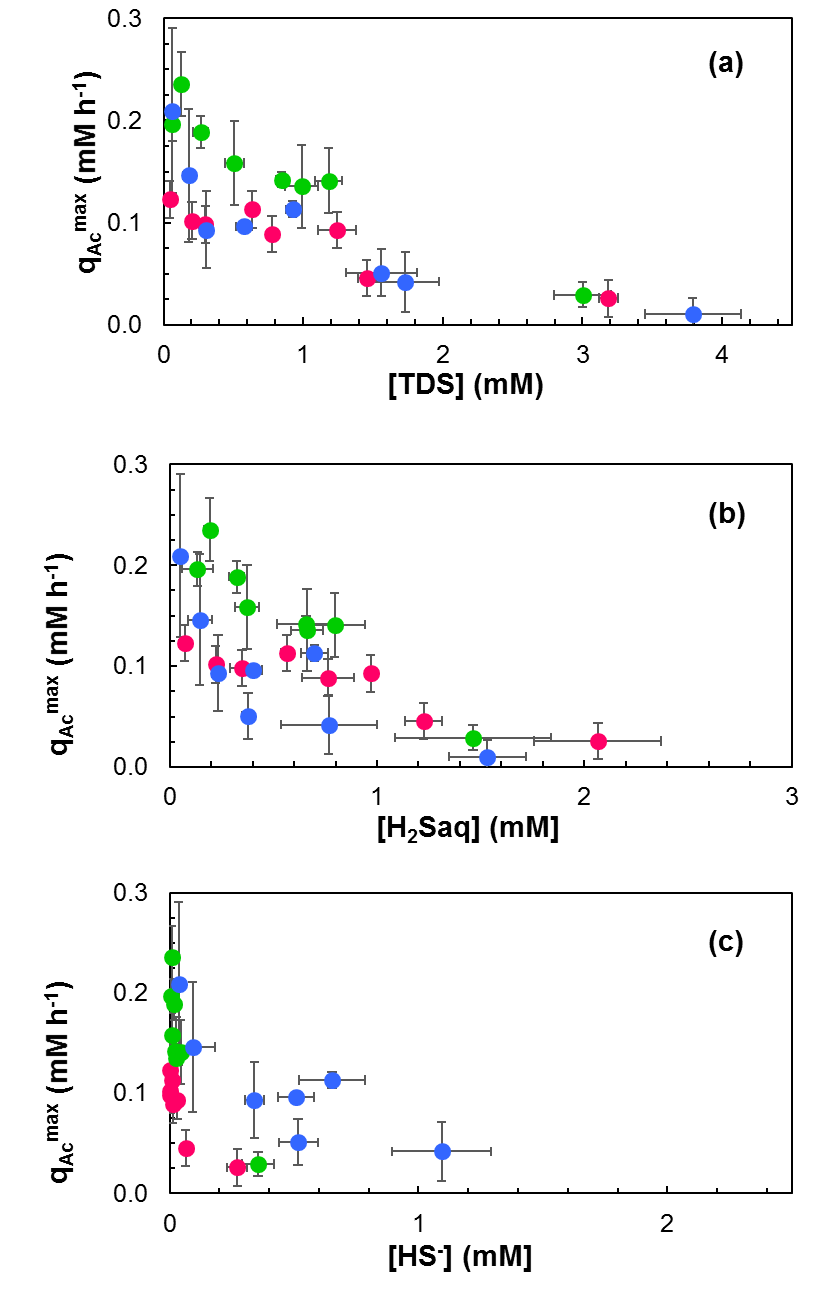


**Fig. S6** – Maximum acetate production rate (mM h^-1^), calculated in a 48 h basis, plotted against: (a) initial total dissolved sulfide concentration ([TDS]) (mM), (b) initial dissolved hydrogen sulfide concentration ([H_2_S_aq_]) (mM) and (c) initial bisulfide concentration ([HS^-^]) (mM) at pH 7 ( ), 6 ( ) and 5 ( ). Data are averages of 3 incubations, error bars represent standard deviations of biological triplicates.


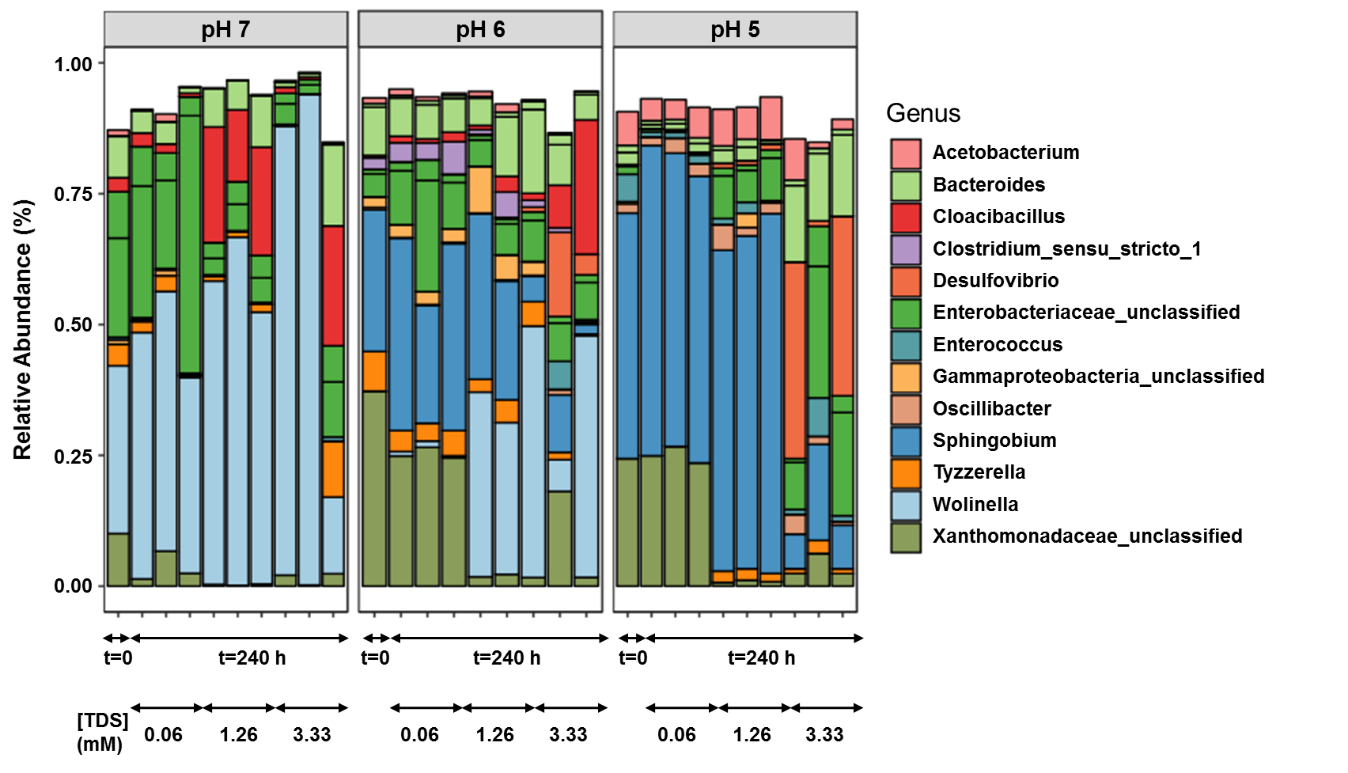


**Fig. S7** - Relative Abundances (%) of the 15 most abundant OTUs, in 3 biological replicates (2 biological replicates at pH 6 and 1.26 mM [TDS]) at t=240h.

**2.1 Clone library results.** Forward sequencies where the genus *Sphingobium* was identified.

>ElefF08.F

AGAGTTTGATCCTGGCTCAGAACGAACGCTGGCGGCATGCCTAATACATGCAAGTCGAACGAACTCTTCGGAGTTAGTGGCGCACGGGTGCGTAACGCGTGGGAATCTACCCAGAGGTTCGGAATAACGGTTGGAAACGACTGCTAATACCGGACGATGTCGAGAGACCAAAGATTTATCGCCTTTGGATGAGCCCGCGTAAGATTAGCTAGTTGGTGAGGTAAAGGCTCACCAAGGCTACGATCTTTAGCTGGTCTGAGAGGATGATCAGCCACACTGGGACTGAGACACGGCCCAGACTCCTACGGGAGGCAGCAGTAGGGAATATTGGACAATGGGGGCAACCCTGATCCAGCAATGCCGCGTGAGTGATGAAGGCCTTAGGGTTGTAAAGCTCTTTTACCCGGGATGATAATGACAGTACCGGGAGAATAAGCTCCGGCTAACTCCGTGCCAGCAGCCGCGGTAATACGGAGGGAGCTAGCGTTGTTCGGAATTACTGGGCGTAAAGCGCACGTAGGCGGGCTATTTAAGTCAGAGGTGAAAGCCCGGGGCTCAACCCCGGAACTGCCTTTGAGACTGGATAGCTTGAATCCTGGAGAGGTGAGTGGAATTCCGAGTGTAGGAGGTG

>ElefF10.F

TACGGCTACCTTGTTACGACTTCACCCCAGTCGCTGATCCCACCGTGGTCGCCTGCCTCTCTTGCGAGTTAGCGCAGCGCCTTCGGGTGAAACCAACTCCCATGGTGTGACGGGCGGTGTGTACAAGGCCTGGGAACGTATTCACCGCGGCATGCTGATCCGCGATTACTAGCGATTCCGCCTTCATGCTCTCGAGTTGCAGAGAACAATCCGAACTGAGACGACTTTTGGAGATTAGCTACCCCTCGCAGGGTTGCTGCCCACTGTAGTCGCCATTGTAGCACGTGTGTAGCCCAACGCGTAAGGGCCATGAGGACTTGACGTCATCCCCACCTTCCTCCGGCTTATCACCGGCGGTTACCTTAGAGTGCCCAACTAAATGATGGCAACTAAGGTCGAGGGTTGCGCTCGTTGCGGGACTTAACCCAACATCTCACGACACGAGCTGACGACAGCCATGCAGCACCTGTCACTTATCCAGCCGAACTGAAGAAAAGCATCTCTGCTAATCACGATAAGGATGTCAAACGTTGGTAAGGTTCTGCGCGTTGCTTCGAATTAAACCACATGCTCCACCGCTTGTGCAGGCCCCCGTCAATTCCTTTGAGTTTTAATCTTGCGACCGTACTCCCCAGGCGGATAACTTAATGCGTTAGCTGCGCCACCCAAGTTCCATGAACCCGGACAGCTAGTTATCATCGTTTACGGCGTGGACTACCAGGGTATCTAATCCTGTTTGCTCCCCACGCTTTCGCACCTCAGCGTCAATACCTGTCCAGTGAGCCGCCTTCGCCACTGGTGTTCTTCCGAATATCTACGAATTTCACCTCTACACTCGGAATTCCACTCACCTCTCCAGGATTCAAGCTATCCAGTCTCAAAGGCAGTTCCGGGGTTGAACCCCGGGCTTTCACCTCTGACTTAAATAGCCGCCTACTGGCGCTTTACTCCCAGGAATTCCGAACAACGCT

>ElefF11.F

AGAGTTTGATCCTGGCTCAGAACGAACGCTGGCGGCATGCCTAATACATGCAAGTCGAACGAACTCTTCGGAGTTAGTGGCGCACGGGTGCGTAACGCGTGGGAATCTACCCAGAGGTTCGGAATAACGGTTGGAAACGACTGCTAATACCGGACGATGTCGAGAGACCAAAGATTTATCGCCTTTGGATGAGCCCGCGTAAGATTAGCTAGTTGGTGAGGTAAAGGCTCACCAAGGCTACGATCTTTAGCTGGTCTGAGAGGATGATCAGCCACACTGGGACTGAGACACGGCCCAGACTCCTACGGGAGGCAGCAGTAGGGAATATTGGACAATGGGGGCAACCCTGATCCAGCAATGCCGCGTGAGTGATGAAGGCCTTAGGGTTGTAAAGCTCTTTTACCCGGGATGATAATGACAGTACCGGGAGAATAAGCTCCGGCTAACTCCGTGCCAGCAGCCGCGGTAATACGGAGGGAGCTAGCGTTGTTCGGAATTACTGGGCGTAAAGCGCACGTAGGCGGCTATTTAAGTCAGAGGTGAAAGCCCGGGGCTCAACCCCGGAACTGCCTTTGAGACTGGATAGCTTGAATCCTGGAGAGGTGAGTGGAATTCCGAGTGTAGAGGTGAAATTCGTAGATATTCGGAAGAACACCAGTGGCGAAGGCGGCTCACTGGACAGGTATTGACGCTGAGGTGCGAAAGCGTGGGGAGCAAACAGGATTAGATACCCTGGTAGTCCACGCCGTAAACGATGATAACTAGCTGTCCGGGTTCATGGAACTTGGGTGGCGCAGCTAACGCATTAAGGTTATCCGACCTGGGG

>ElefF16.F

TACGGTTACCTTGTTACGACTTCACCCCAGTCGCTGATCCCACCGTGGTCGCCTGCCTCTCTTGCGAGTTAGCGCAGCGCCTTCGGGTGAAACCAACTCCCATGGTGTGACGGGCGGTGTGTACAAGGCCTGGGAACGTATTCACCGCGGCATGCTGATCCGCGATTACTAGCGATTCCGCCTTCATGCTCTCGAGTTGCAGAGAACAATCCGAACTGAGACGACTTTTGGAGATTAGCTACCCCTCGCAGGGTTGCTGCCCACTGTAGTCGCCATTGTAGCACGTGTGTAGCCCAACGCGTAAGGGCCATGAGGACTTGACGTCATCCCCACCTTCCTCCGGCTTATCACCGGCGGTTACCTTAGAGTGCCCAACTAAATGATGGCAACTAAGGTCGAGGGTTGCGCTCGTTGCGGGACTTAACCCAACATCTCACGACACGAGCTGACGACAGCCATGCAGCACCTGTCACTTATCCAGCCGAACTGAAGAAAAGCATCTCTGCTAATCACGATAAGGATGTCAAACGTTGGTAAGGTTCTGCGCGTTGCTTCGAATTAAACCACATGCTCCACCGCTTGTGCAGGCCCCCGTCAATTCCTTTGAGTTTTAATCTTGCGACCGTACTCCCCAGGCGGATAACTTAATGCGTTAGCTGCGCCACCCAAGTTCCATGAACCCGGACAGCTAGTTATCATCGTTTACGGCGTGGACTACCAGGGTATCTAATCCTGTTTGCTCCCCACGCTTTCGCACCTCAGCGTCAATACCTGTCCAGTGAGCCGCCTTCGCCACTGGTGTTCTTCCGAATATCTACGAATTTCACCTCTACACTCGGAATTCCACTCACCTCTCCAGGATTCAAGCTATCCAGTCTCAAAGGCAGTTCCGGGGTTGAGCCCCGGGCTTTCGCCTCTGACTTAAATAAGCCGCCTACGTGCGCTTTACGCCCAGTAATTCCGAACAACGCTAGCTCCCTCCGTATTACCGCGGCTGCTG

**References**

Folens, K., Van Acker, T., Bolea-Fernandez, E., Cornelis, G., Vanhaecke, F., Du Laing, G. & Rauch, S. (2018) Identification of platinum nanoparticles in road dust leachate by single particle inductively coupled plasma-mass spectrometry. *Science of the Total Environment* **615**: 849–856.

Klindworth, A., Pruesse, E., Schweer, T., Peplies, J., Quast, C., Horn, M. & Glöckner, F.O. (2013) Evaluation of general 16S ribosomal RNA gene PCR primers for classical and next-generation sequencing-based diversity studies. *Nucleic Acids Research* **41**: 1–11.

Kozich, J.J., Westcott, S.L., Baxter, N.T., Highlander, S.K. & Schloss, P.D. (2013) Development of a Dual-Index Sequencing Strategy and Curation Pipeline for Analyzing Amplicon Sequence Data on the MiSeq Illumina Sequencing Platform. *Applied and Environmental Microbiology* **79**: 5112–5120.

Patil, S.A., Arends, J.B.A., Vanwonterghem, I., Meerbergen, J. Van, Guo, K., Tyson, G.W. & Rabaey, K. (2015) Selective enrichment establishes a stable performing community for microbial electrosynthesis of acetate from CO2. *Environmental Science and Technology* **49**: 8833 – 8843.

Sander, R. (2015) Compilation of Henry’s law constants ( version 4 . 0 ) for water as solvent. *Atmospheric Chemistry and Physics* **15**: 4399–4981.

Schloss, P.D., Gevers, D. & Westcott, S.L. (2011) Reducing the effects of PCR amplification and sequencing artifacts on 16S rRNA-based studies. *PLoS ONE* **6**: e27310.

Wang, Q., Garrity, G.M., Tiedje, J.M. & Cole, J.R. (2007) Naïve Bayesian Classifier for Rapid Assignment of rRNA Sequences into the New Bacterial Taxonomy. *Applied and Environmental Microbiology* **73**: 5261–5267.
